# Landscape and flux for quantifying global stability and dynamics of game theory

**DOI:** 10.1371/journal.pone.0201130

**Published:** 2018-08-08

**Authors:** Li Xu, Jin Wang

**Affiliations:** 1 State Key Laboratory of Electroanalytical Chemistry, Changchun Institute of Applied Chemistry, Chinese Academy of Sciences, Changchun, Jilin, China; 2 Department of Chemistry and Physics, State University of New York at Stony Brook, Stony Brook, NY, United States of America; Southwest University, CHINA

## Abstract

Game theory has been widely applied to many research areas including economics, biology and social sciences. However, it is still challenging to quantify the global stability and global dynamics of the game theory. We developed a landscape and flux framework to quantify the global stability and global dynamics of the game theory. As an example, we investigated a model of three-strategy game: a special replicator mutator game termed as the repeated Prison Dilemma model. In this model, one stable state, two stable states and limit cycle can emerge under different parameters. The repeated Prisoner’s Dilemma system has Hopf bifurcation from one stable state to limit cycle state, and then to another one stable state or two stable states, and vice versa. We quantified the global stability of the repeated Prisoner’s Dilemma system and identified optimal kinetic paths between the basins of attractor. The optimal paths are irreversible due to the non-zero flux. We also quantified the interplay between *Peace* and *War*.

## Introduction

Game theory is the study of conflict and cooperative strategic decision making between intelligent rational decision-makers. Game theory has widely been recognized to be important and useful in many fields such as economics, political science, psychology, computer science, biology etc. The game dynamics can usually converge to stable point attractors [1, 2]. However, a more complex dynamics can emerge as stable oscillations. The cyclical oscillations have been explored in the game-theoretical models of price dispersion [3]. The cyclical oscillation has also been found in the side-blotched lizard with three color morphs in male signalling. They played three different alternative reproductive strategies(Uta stansburiana) [4]. Even though numerous studies have been explored over the past decades with great advances in this field, there are still several unsolved problems. But understanding the underlying the global dynamics and global stability of the game theory is still one of the greatest challenges at present.

The evolutionary stability was first introduced and formulated by Maynard Smith and Price in 1973 [5, 6]. They first applied the game theoretical ideas to evolutionary biology and population dynamics. This is the birth of evolutionary game theory which studies the behaviors of the large populations [2]. Evolutionary population game is a general framework for exploring the strategic interactions among large populations of agents. The agents play pure strategies with random matching. The significant applications of the game theory are on modeling and analyzing varieties of human and animal behaviors around us. The game theory systems involve different interactions among the agents. The strategic interactions can lead to complex dynamics. The dynamics of biological structure and population behaviors often have high stability and robustness. Thus, one of the central problems is how to explore the global stability and robustness of the evolutionary game theory in a population.

There have been many studies on the stability of game theory [2, 6, 7]. However, most of the investigations are focused on the local stability. The purpose of local stability analogs is to uncover whether a system can restore to equilibrium under a small disturbance. An evolutionary stable strategy (ESS) is a strategy which is resistent to the invasions by a few mutants playing a different strategy in a population [6]. The system can move far from its ESS equilibrium since it is under continuous small perturbations from the mutations and the events by chance [7]. Thus, the ESS can not guarantee the global stability of the system. A Nash equilibrium (NE) is a result of a non-cooperative game [6]. Each player is assumed to know the other players’ equilibrium strategy and the players gain nothing by altering only their own strategy. The NE can be stable or unstable. It is very similar to an ESS [6]. The evolutionary stable strategy (ESS) and Nash equilibrium (NE) are insufficient conditions of dynamic stability since they are only local criterions under small fuctuations [7]. It is often not clear whether the system will reach equilibrium from an arbitrary initial state or whether the system can be switched from one locally stable state to another. These dynamical issues depend on the global structure of the system. Furthermore, the link between the global characterization and the dynamics of the game theory systems is often lacking understanding the global stability of the game theory is thus still challenging at present.

Deterministic population dynamics can only describe the average dynamics of the system. Both external and intrinsic fluctuations are unavoidable [8]. The environmental fluctuations can influence the behaviors of population. The intrinsic fluctuations originated from mutations or random errors in implementations, can not be neglected in finite population. They may play an essential role in the dynamics of the system. The stochastic evolutionary game dynamics was first studied by Foster and Young in 1990 [7]. They defined the stochastic stability and the stochastically stable set which is a set of stochastically stable equilibrium states. It is assumed that the fluctuations approach to zero slowly in the long run(so called long-run equilibria) [7, 9]. The stable state sets can be obtained by the potential theory [7]. However, the general approach for exploring the global stability of the game theory systems are still absent.

The researchers have also explored the game theory system with the method of Lyapunov function which can be used to study the global stability [7, 9]. Certain analytical Lyapunov functions were found for some highly simplified game theory models [2]. However, it is still challenging to find the Lyapunov function for the general game theory with complex dynamics. In this study, we will provide a general approach to investigate the Lyapunov function. We will also develop a general framework for exploring the robustness and the global stability of the game theory systems.

In addition to the dynamics with simple convergent stable states, exploring the mechanism of the non-convergent behavior is even more important for understanding the nature of the dynamics for evolutionary game theory. This is because certain more complicated behaviors such as oscillations and chaos often emerge in real biological interactions [10]. The most well known model of evolutionary dynamics is the replicator model. The simplest replicator dynamics of three-strategy games can give arise to certain behaviors: sinks, sources and saddles or heteroclinic cycles for Rock-Paper-Scissors(RPS) game [10, 11]. However, the replicator dynamics can not provide a stable limit cycle behavior [10, 12]. On the other hand, Lyapunov stable equilibria of the replicator dynamics are Nash equilibria and ESS which are asymptotically stable [13, 14]. Mutation effects can be included in order to promote the chances that players change from one strategy to another spontaneously. The selection and mutation model has been explored in population genetics for decades [6]. The replicator-mutator dynamics plays a key role in evolutionary theory [6, 13–16].

In this study, we will develop a non-equilibrium landscape and flux framework to quantify the global stability and robustness of evolutionary game theory. Conventional stability analysis of game theory (Nash equilibrium and ESS) only provieds a static view and local description. We will give a dynamical view and global quantification of the stability. We found the flux is an additional force as a signature and quantitative measure of the degree of detailed balance breaking or nonequilibriumness, which is not present in determining the global dynamics of the conventional game theory. Both landscape and flux determines the game dynamics. The landscape topography and kinetic transitions as well as optimal kinetic paths from one basin to another (local stable strategy) can provide the global quantification of the strategy switching process and functional stability. This is also not present in the current game theory. The global stability can also be systematically studied with the landscape and flux approach via Lyapunov functions. It is worth noticing that the Lyapunov functions are only found for special cases (one dimensional case) in the current game theory [7]. In present evolutional game theory, the driving forces have never been decomposed. The landscape and flux theory provides a framework to quantify each component of the driving forces and describe the global evolutionary game dynamics. We also explored non-equilibrium thermodynamics which is not covered in current game or evolutionary game theory.

The prisoner’s dilemma is a famous example in game theory [2, 6]. Two players might not cooperate, even though more profit can be earned if they cooperate. For example, the two gang members are arrested into a prison. They are not allowed to communicate with each other. The prosecutors lack sufficient evidence to convict them of a crime. Both Gang member A and gang member B will be in prison have 2 years if each betrays the other. Gang member A will be free and gang member B will be in prison for 3 years (and vice versa) if gang member A betrays member B but gang member B remains silent. Gang member A and gang member B will only be in prison for 1 year if both keep silent. This shows that betraying gains a greater reward than cooperation. Therefore, the two gang members tend to betray each other to gain more profits from their own consideration [2, 6]. In reality, human beings often choose a cooperative strategy as keeping silent to avoid losing more profits. This can lead to a win-win situation. However, since both game players make decisions for the goals of gaining more profits, the win-win scenario for the best profit may not be realized in real life. In fact, this is common in public resource development and utilization, the market competition, and the environmental issues [2, 6]. Therefore, when everyone is trying to maximize his or her own benefits, the profits gained from both sides are impaired. This is also a common situation in microeconomics when everyone maximizes his or her own benefit. This phenomenon challenges the conventional thinking. It raises some interesting questions such as how to avoid the prisoner’s dilemma, cooperate, abide by the agreement and so on. Thus, personal best interest maximization is not necessarily the best strategy in an interactive world [2, 6]!

**Fig 1:**
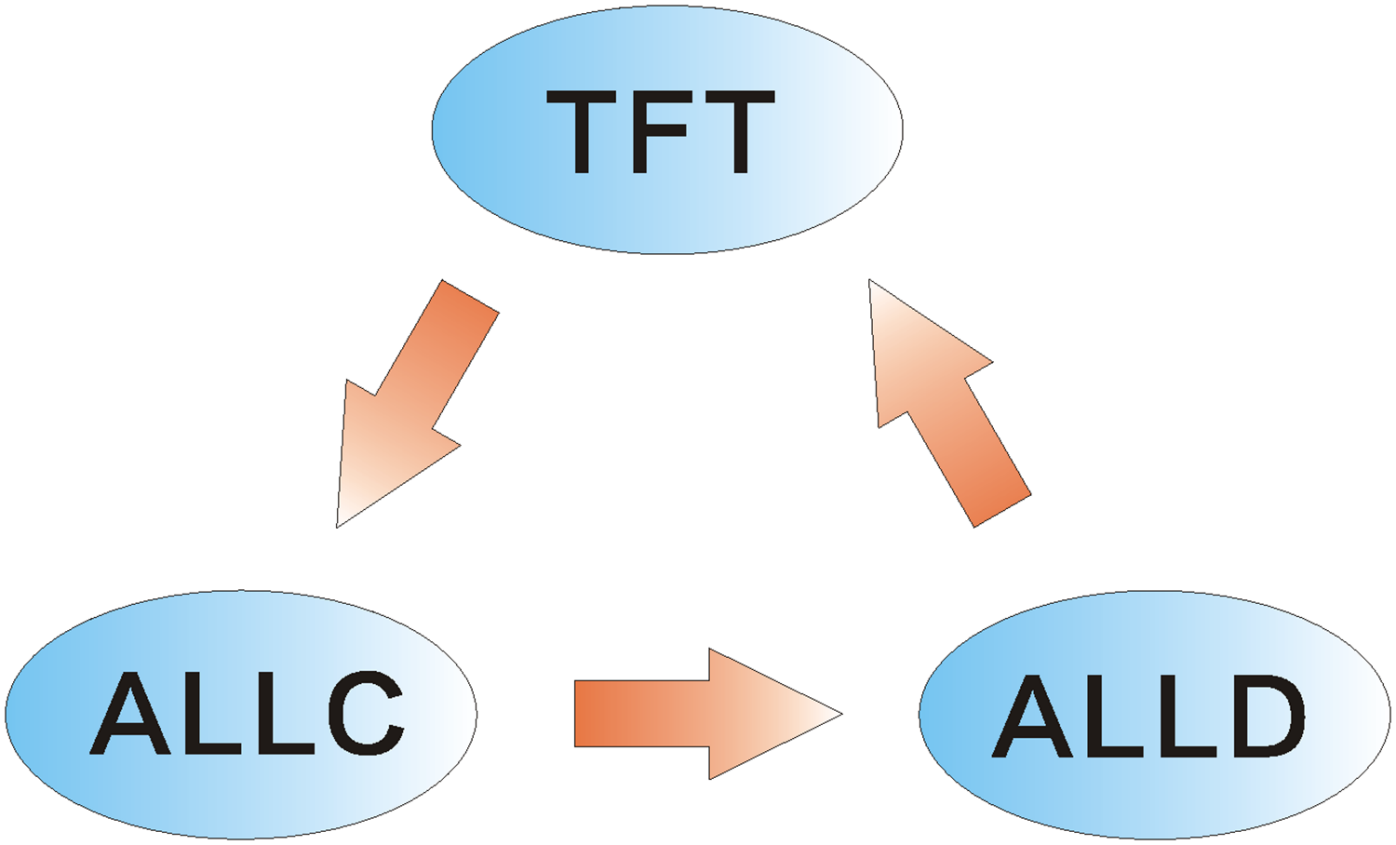
The schematic of repeated prisoner’s dilemma. *ALLC* is short for always cooperate. *ALLD* is short for always defect. *TFT* is short for tit-for-tat.

We use a representative Prisoner’s Dilemma model-the repeated Prisoner’s Dilemma model [13, 17–19] as an example to illustrate our general theory. There are three interactional strategies in this model: always cooperate simplified by *ALLC*; always defect simplified by *ALLD*; and tit-for-tat simplified by *TFT*. Fig 1 shows the schematics of repeated Prisoner’s Dilemma model. *ALLD* players are the first winners with random initial strategies in the population. Then small numbers of *ALLC* players will invade and replace strategy *ALLD*. The consideration of mutation effect can lead the repeated Prisoner’s Dilemma to a stable limit cycle state rather than a *ALLD* dominant state, even though *ALLD* is the only strict Nash equilibrium [6]. There existed sustained oscillations among *ALLD*, *ALLC* and *TFT* strategies in the repeated Prisoner’s Dilemma model. We developed a landscape and flux landscape theory [20–26] to explore the global behavior and the dynamics of the repeated Prisoner’s Dilemma game system. We quantified the population landscape related to the steady state probability distribution. It can determine the global behavior of the system. The landscape has the attractor basins for multi-stability games and Mexican hat for limit cycle oscillations games. We also found the intrinsic landscape with a Lyapunov function feature of the repeated Prisoner’s Dilemma game model. It can be used to quantify the global stability of the system. The non-equilibrium evolutionary game theory dynamics for repeated Prisoner’s Dilemma model is found to be determined by both the landscape and the curl flux. The curl steady state probability flux can result in the break down of the detailed balance and drive the stable periodical oscillation flow along the limit cycle ring [20]. We also explored the stability and robustness of the repeated Prisoner’s Dilemma game against the mutation rate and the pay-off matrix. The optimal kinetic pathways between basins are quantified by the path integral method and irreversible due to the non-zero flux.

## Materials and methods

### The landscape and flux quantification for game theory

The landscape and flux theory as well as the non-equilibrium thermodynamics for the general dynamical systems have been explored in several different fields [20–27]. They can be used to address the issues of global stability, function, robustness for dynamical systems. Here, we will apply the landscape and flux theory to quantify the global stability and robustness of the game theory.

We consider a large finite population of players who play a finite set of pure strategies with random matching. Each player chooses a pure strategy from the strategy set *S* = 1, 2, 3, …, *n* [1, 2]. The aggregated behaviors of these players are described by a population state **x**, with *x*_*i*_ representing the proportion of players choosing pure strategy *S*_*i*_. And *x*_*i*_ represents the frequency of strategy *S*_*i*_. Due to the intrinsic and the extrinsic fluctuations [8], the deterministic dynamics described by a set of ordinary differential equations are supplemented with the additional fluctuation force. Then the stochastic dynamics emerges [28, 29]: *d***x** = **F**(**x**)*dt* + **g** ⋅ *d***W**, where **x** is the state vector representing the strategy density in game dynamics), **F**(**x**) is the driving force, **W** as a vector coupled through the matrix *g* represents an independent Wiener process. The evolution of the stochastic dynamics is thus more appropriately described by the probability evolution. The probability distribution *P*(**x**, *t*) in time can be obtained by solving the corresponding Fokker-Planck diffusion evolution equation [28, 29]:

$$\partial P/\partial t=-\nabla \cdot J=-\nabla \cdot [FP-\left(1/2\right)\nabla \cdot \left(\left(g\cdot g^{T}\right)P\right)].$$ (1)

We set *D***D** = (1/2)(**g** ⋅ **g**^**T**^), where *D* is a constant describing the scale of the fluctuations and **D** represents the anisotropy diffusion matrix of the fluctuations. The Fokker-Plank diffusion equation describes the game dynamics as a conservation law of probability. The change in local probability is equal to the net flux in or out.

The game process can be treated as a binomial sampling process: *N* players play the games with different strategies from a large population of players, just like *n* alleles in a diploid population of constant size *N* [24]. So we can set *D*_*ij*_ = *x*_*i*_(*δ*_*ij*_ − *x*_*j*_) coming from the sampling nature of the game which is widely used in evolutionary population dynamics [24].

The matrix *D*_*ij*_ has some special features [24]. The first is

$${\left(\nabla \cdot D\right)}_{i}=1-nx_{i},$$ (2)

so that $\sum_{i=1}^{n}{\left(\nabla \cdot D\right)}_{i}=0$. The second is its inverse matrix is known to have the feature [24]:

$${\left(D^{-1}\cdot F\right)}_{i}=F_{i}/x_{i}-F_{n}/x_{n},$$ (3)

where $F_{n}=-\sum_{i=1}^{n-1}F_{i}$.

The steady state probability distribution *P*_*ss*_ can be derived from the long time limit of the Fokker-Planck equation ∂*P*/∂*t* = 0. The steady state probability flux is defined as **J**_**ss**_ = **F***P*_*ss*_ − *D*∇ ⋅ (**D***P*_*ss*_). The steady state flux is divergent free from Eq 1 and therefore a rotational curl. The population landscape is defined as *U* = − ln*P*_*ss*_. Then, the deterministic driving force **F** can be decomposed as: **F** = −*D***D** ⋅ ∇*U* + **J**_*ss*_/*P*_*ss*_ + *D*∇ ⋅ **D**. The flux **J**_*ss*_ = 0 denotes the equilibrium with detailed balance since net flux is zero while the net flux **J**_*ss*_ ≠ 0 measures the degrees of detailed balance breaking and therefore the degree of one-equilibriumness. Therefore, the dynamics of the equilibrium game system is determined only by the population landscape gradient. The dynamics of the non-equilibrium system is determined by both the potential landscape and non-zero flux. [20]

The intrinsic landscape at the zero fluctuation limit has the the feature of the Lyapunov function [24, 30–32] and can be used to quantify the global stability and function of the game theory systems. The intrinsic landscape *ϕ*_0_ follows the Hamilton—Jacobi equation as below [23, 24, 33]:

$$F\cdot \nabla \phi_{0}+\nabla \phi_{0}\cdot D\cdot \nabla \phi_{0}=0.$$ (4)

and

$$\frac{d\phi }{dt}=F\cdot \nabla \phi_{0}=-\nabla \phi_{0}\cdot D\cdot \nabla \phi_{0}\leq 0$$ (5)

As seen, the intrinsic landscape *ϕ*_0_ is a Lyapunov function monotonically decreasing along a deterministic path for the game dynamics [24, 33]. The intrinsic landscape *ϕ*_0_ quantifies the global stability for general dynamical systems including game dynamics either with or without detailed balance and can be solved by the level set method [34].

The steady state probability distribution *P*_*ss*_ and the population landscape *U* have the relationship of *P*_*ss*_(**N**) = exp(−*U*)/*Z*. *Z* is the partition function *Z* = *∫* exp(−*U*)*d***N**. The entropy of the non-equilibrium game system is given as [24, 35–38]: *S* = −*∫* *P*(**N**, *t*)ln*P*(**N**, *t*)*d***N**. The energy can be defined as: *E* = *D* *∫* *UP*(**N**, *t*)*d***N** = −*D* *∫* ln[*ZP*_*ss*_]*P*(**N**, *t*)*d***N**. Thus, the nonequilibrium free energy F is:

$$F=E-DS=D(\int P\;\text{ln}\left(P/P_{ss}\right)\;dN-\text{ln}Z).$$ (6)

The nonequilibrium free energy as the combination of energy and entropy reflects the first law of non-equilibrium thermodynamics. The free energy decreases in time monotonically while reaching its minimum value, F=-DlnZ [24, 35–38]. This reflects the second law of non-equilibrium thermodynamics. The free energy as a Lypunov functional can be used to explore the global stability of the stochastic non-equilibrium systems with finite fluctuations.

Game theory systems are often non-equilibrium open systems. They often exchange energy and information from the environments. This leads to dissipation. The evolution of the entropy in time can be separated into two terms [20, 24, 39, 40]: $\dot{S}={\dot{S}}_{t}-{\dot{S}}_{e}$, where ${\dot{S}}_{t}=\int dx\left(J\cdot {\left(DD\right)}^{-1}\cdot J\right)/P^{2}$ is entropy production rate which is positive or zero, and ${\dot{S}}_{e}=\int dx\left(J\cdot {\left(DD\right)}^{-1}\cdot F^{\prime }\right)/P$ is heat dissipation rate which represents the entropy flow rate to the non-equilibrium system from the environments. It can be either positive or negative. The effective force is shown as **F**′ = **F** − *D*∇ ⋅ **D**. $\dot{S}$ can be interpreted as the entropy change of the non-equilibrium system, and the ${\dot{S}}_{t}$ can be interpreted as the total entropy change of the system and environments. ${\dot{S}}_{t}$ is always non-negative according with the thermodynamic second law. The entropy production rate and heat dissipation rate are equal in steady state [20, 24, 39, 40]. Thus, the entropy production rate is another global physical characterization of a non-equilibrium system.

### The repeated Prisoner’s Dilemma game theory model with mutations

We set **A** as the payoff matrix [6, 13–16]. The scalar *A*_*i*_(**x**) represents the payoff to strategy *S*_*i*_ when the population state is represented by **x**. Since the sum of all frequencies is equal to 1: ∑_*i*_ *x*_*i*_ = 1, the system becomes *n* − 1 dimensional. The average fitness (pay-off) $\bar{f}$ is obtained as $\bar{f}=\text{xAx}$ by the players of the population [1, 2, 6]. The fitness denotes the individual’s evolutionary success [9]. In the game theory, the payoff of the game is the fitness. The fitness to strategy *i* becomes *f*_*i*_ = (**Ax**)_**i**_ [1, 2]. In this study, we use simple three-strategy game which can be reduced to two dimensional dynamics.

The players in standard Prisoner’s Dilemma model play either cooperate strategy or defect strategy simultaneously [41]. The players earn their payoff depending on their choices of the strategies. The mutually aided cooperators will acquire the reward *R* when the cooperators are encountered. The defectors will obtain a punishment *Pu* when the defectors are encountered. A cooperator acquires a sucker payoff *S* and a defector acquires a temptation payoff *T* when they encountered [18, 42]. The payoff matrix of the Prisoner’s Dilemma model should satisfy the relationship *T* > *R* > *Pu* > *S*. Mutual cooperative strategy is better than mutual defective strategy, so the reward *R* should be greater than the punishment *Pu*. Defectors gain more temptation *T* than the reward *R* that cooperators gain if the partner cooperates. The defectors will lose less punishment *Pu* than the lost sucker *S* of cooperators if the partner defects.

We explored a repeated Prisoner’s Dilemma game model with mutation using the replicator dynamics. Replicator dynamics was introduced by Taylor and Jonker (1978) [43], which is the best-known dynamics in the models of biological evolution. The mean dynamic evolutionary equation is the replicator dynamics shown as below [1, 2]

$$\frac{dx_{i}}{dt}=x_{i}\left(f_{i}-\bar{f}\right)=x_{i}\left({\left(\text{Ax}\right)}_{i}-\text{xAx}\right)$$ (7)

In the presence of mutations, *ALLD* and *ALLC* players may mutate to strategy *TFT*. Therefore, more players choose *TFT* strategy, and help *ALLC* players to win the game. Then the *ALLD* players will emerge again to obtain more profit [6]. This dynamics can be used to quantify the peace and war oscillation [6].

We define the probability that agents with strategy *S*_*i*_ mutating to strategy *S*_*j*_ as *q*_*ij*_, which satisfies ∑_*j*_ *q*_*ij*_ = 1. Thus, mutation matrix is *Q* = [*q*_*ij*_] [6, 13, 17, 19]. The elements of the mutation matrix *Q* are defined in terms of a mutation parameter *μ* satisfying 0 ≤ *μ* ≤ 1. The mutation *μ* denotes the error probability in the process of replication. *μ* = 0 denotes no mutation with perfect replication while *μ* = 1 denotes the mutation completely. The replicator-mutator dynamics describes the dynamics of the population distribution **x** as a result of replication driven by fitness **f** and mutation driven by *Q* [6, 15, 16]:

$$\frac{dx_{i}}{dt}=\sum_{j=1}^{N}x_{i}f_{i}\left(x\right)q_{\text{ji}}-x_{i}\bar{f}$$ (8)

This equation is the quasi-species equation proposed by Manfred Eigen and Peter Schuster [6, 15, 16]. We set a uniform probability of mutation from one strategy to another strategy with *q*_*ii*_ = 1 − 2*μ* which shows the players with the same strategy can get profits 1 − 2*μ* for each player, and *q*_*ij*_ = *μ* which shows the player chooses strategy *i* will get profits *μ* when he encounters the player with strategy *j*. Then the matrix *Q* can be shown as follows [6, 15, 16]:

$$Q=(\begin{array}{ccc} 1-2\mu & \mu & \mu \\ \mu & 1-2\mu & \mu \\ \mu & \mu & 1-2\mu \end{array})$$ (9)

Let *x*_1_, *x*_2_, *x*_3_ represent the fractions of the population choosing the strategies *ALLD*, *TFT*, *ALLC*, respectively. We substitute *Q* into Eq 8, then the replicator-mutator dynamics are shown as the following simplified equations [18, 42]:

$$\begin{array}{c} dx_{1}/dt=x_{1}\left(f_{1}-\bar{f}\right)+\mu \left(-2x_{1}f_{1}+x_{2}f_{2}+x_{3}f_{3}\right)\\ dx_{2}/dt=x_{2}\left(f_{2}-\bar{f}\right)+\mu \left(-2x_{2}f_{2}+x_{1}f_{1}+x_{3}f_{3}\right)\\ dx_{3}/dt=x_{3}\left(f_{3}-\bar{f}\right)+\mu \left(-2x_{3}f_{3}+x_{1}f_{1}+x_{2}f_{2}\right) \end{array}$$ (10)

We considered the players playing with infinite number of rounds. So in these limits, the average payoff matrix with the cost for the strategies *ALLD*, *TFT*, *ALLC* are shown as [17, 18]:

$$A=(\begin{array}{ccc} Pu & Pu & T\\ Pu-c & R-c & R-c\\ S & R & R \end{array})$$ (11)

The mutually aided cooperators will acquire the reward *R* when the cooperators are encountered. The defectors will obtain a punishment *Pu* when the defectors are encountered. A cooperator acquires a sucker payoff *S* and a defector acquires a temptation payoff *T* when they encountered [18, 42]. And we set there is a small complexity cost *c* to playing strategy *TFT* [18, 42].

*TFT* strategy is conditional while *ALLD* and *ALLC* strategies are unconditional. Thus, the payoff value of strategy *TFT* may have a small complexity cost [17, 44].

The parameters are set as: *T* = 5,*R* = 3,*Pu* = 1, *S* = 0 [6, 18, 45]. *μ* is the average mutation rate between each two of the three strategies. *c* is a complexity cost for playing *TFT*. Larger *c* represents less players who play the strategy *TFT*.

## Results

### Phase diagram, Hopf bifurcations and the global stability of evolutionary game dynamics upon *TFT* cost changes

**Fig 2:**
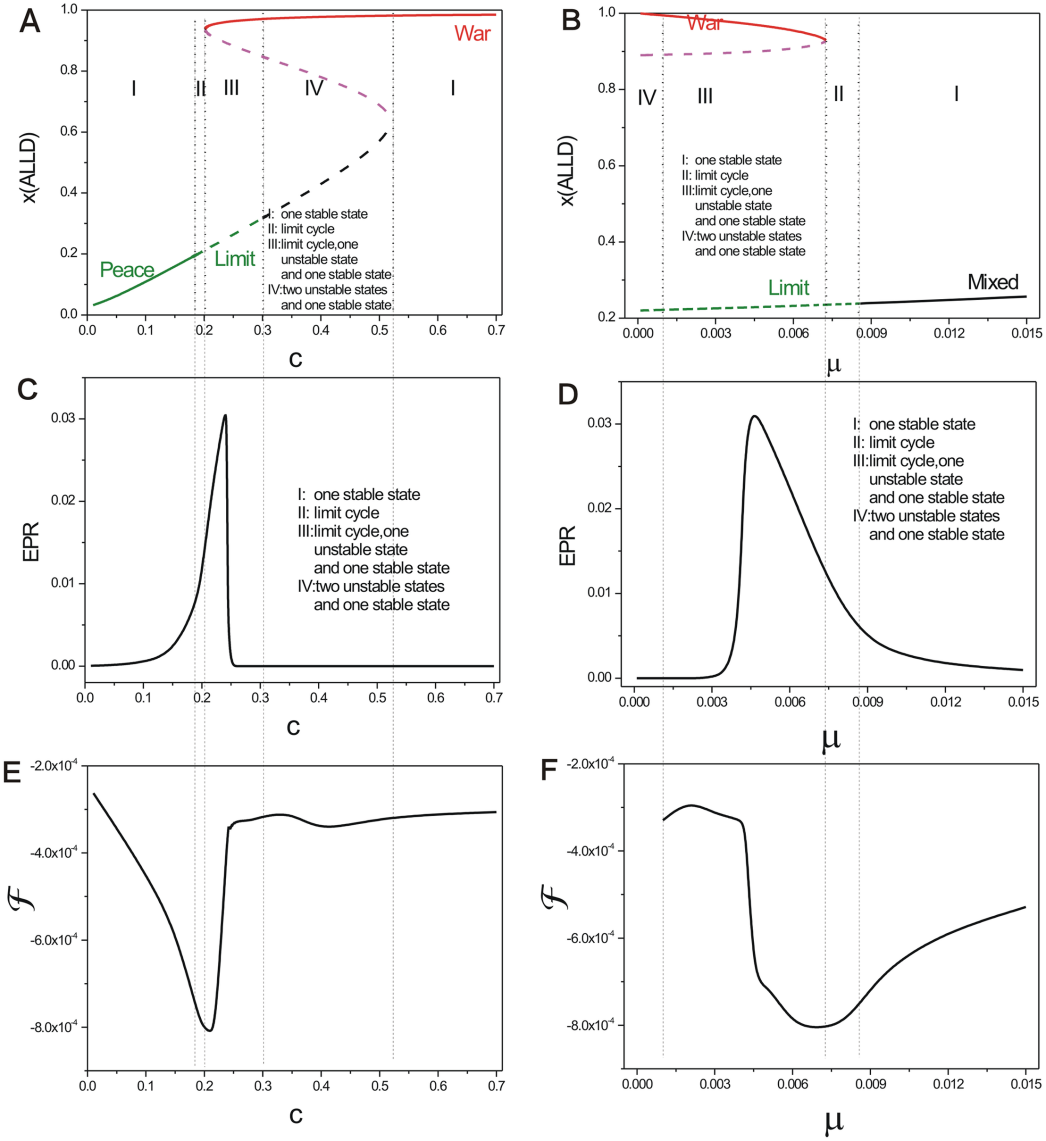
Phase diagram, entropy production rate, and free energy versus different parameter *c*, *μ*. A: the phase diagram for repeated Prisoner’s Dilemma model with the constant parameter *μ* = 0.006 and changing cost parameter *c*. C: the entropy production rate versus parameter *c*. E: the free energy versus parameter *c*. B: the phase diagram for the repeated Prisoner’s Dilemma model with the constant parameter *c* = 0.22 and changing parameter *μ*. D: the entropy production rate versus parameter *μ*. F: the free energy versus parameter *μ*. The other parameters are *T* = 5, *R* = 3, *Pu* = 1, *S* = 0.

Fig 2(A) shows the phase diagram which shows a *S* shape for the repeated Prisoner’s Dilemma with the constant parameter *μ* = 0.006 and changing parameter *c*. There are five regions in this phase diagram. When the cost for *TFT* *c* is smaller, the game theory system has only one stable state, which denotes the *Peace* state in the peace and war game shown in the left Region I. As *c* increases, the stable state becomes a limit cycle in Region II. Then, as *c* increases further, another new stable state (can be viewed as *War* state) and an unstable saddle state emerge beside the limit cycle. This is a saddle-node bifurcation shown in Region III. As cost *c* increases furthermore, the limit cycle diminishes and becomes an unstable state, along with a stable state (*War* state) in Region IV. As cost *c* increases even further, there is only one stable state (*War* state) in the right Region I.

It is interesting to note that the two-phase dynamics of p53 in the DNA damage response was intensively explored by Zhang et al [46]. They revealed that a sequential predominance of distinct feedback loops can lead the system to multiple-phase dynamical behaviors [46]. This shows another similar behavior as our game theory model where the variation of the parameters can also lead the system to multiple-phase dynamical behaviors. It is clear that the environmental changes and internal variations can lead the system to phase transitions.

Fig 2C shows the entropy production rate (EPR) versus cost c. We can see EPR has a bell shape in Region II and Region III under the limit cycle behavior of the system. It indicates that the limit cycle costs more energy to maintain its coherent oscillation.

**Fig 3:**
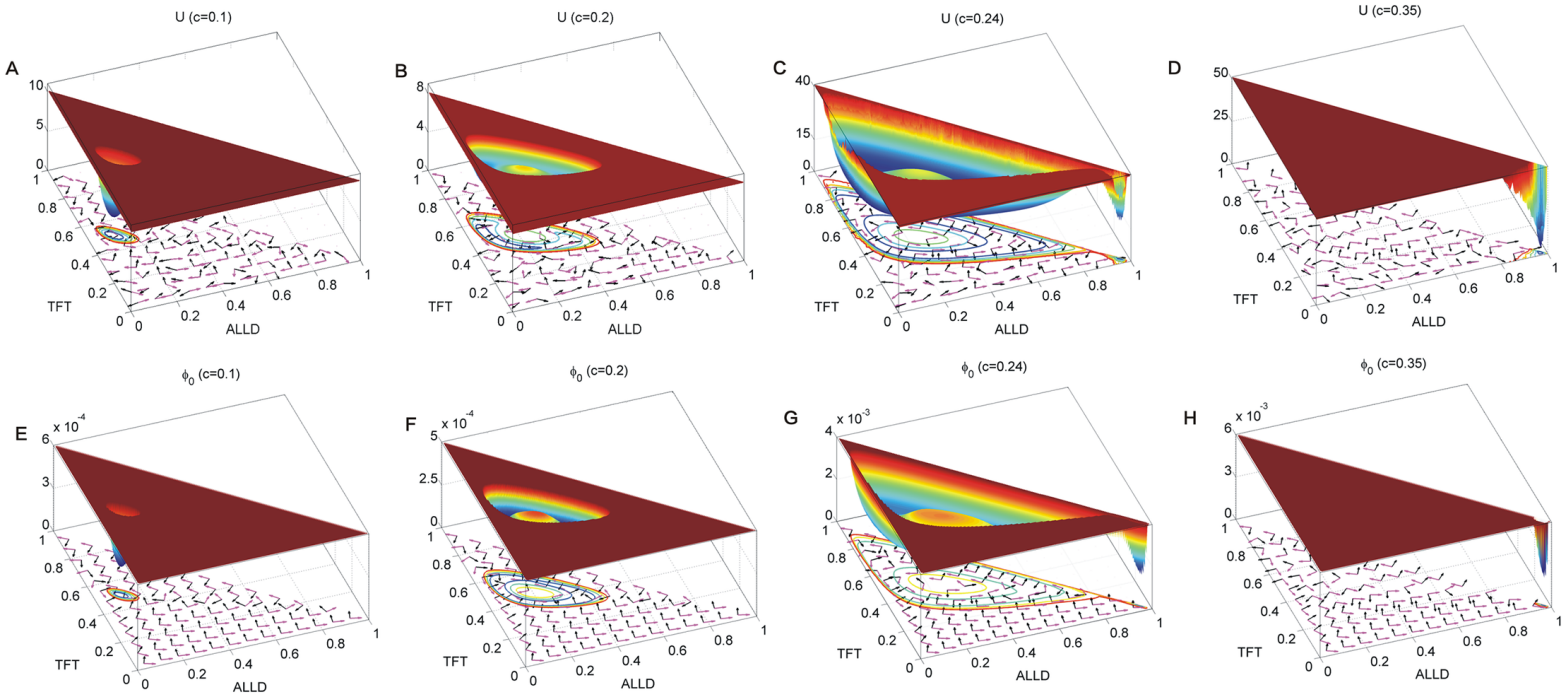
The 3 dimensional population landscapes and The 3 dimensional intrinsic energy landscapes. The 3 dimensional population landscapes *U* with increasing parameter *c* are shown in *A*, *B*, *C*, *D*. Purple arrows represent the flux velocity(**J**_*ss*_/*P*_*ss*_) while the black arrows represent the negative gradient of population potential(−∇*U*). The 3 dimensional intrinsic energy landscapes *ϕ*_0_ with increasing parameter *c* are shown in E, F, G, H. Purple arrows represent the intrinsic flux velocity (**V** = (**J**_*ss*_/*P*_*ss*_)_*D*→0_) while the black arrows represent the negative gradient of intrinsic potential(−∇*ϕ*_0_).

By solving the Fokker-Planck diffusion equation, we obtain the steady distribution of the probability. Thus, the population landscape of the game system can be obtained as: *U* = −*lnP*_*SS*_. We solved the Hamilton-Jacobi equation to obtain the intrinsic landscape *ϕ*_0_ by the level set method [34]. Fig 3 shows the 3 dimensional non-equilibrium population landscape *U* at the top row and intrinsic potential landscape *ϕ*_0_ at the bottom row for the repeated Prisoner’s Dilemma game dynamics as the parameter *c* increases, when the other parameters are set as *μ* = 0.006, *T* = 5, *R* = 3, *Pu* = 1, *S* = 0, *D* = 5 × 10^−4^. We can see the underlying population landscape and the intrinsic landscape have similar shapes. The population landscape and the intrinsic landscape both have a basin shown in Fig 3(A) and 3(E) with small cost *c* = 0.1. Fig 3(B) shows the population landscape *U* with a closed inhomogeneous Mexican hat ring valley which is not uniformly distributed, while Fig 3(F) shows that the intrinsic landscape *ϕ*_0_ with Lyapunov feature has a closed homogeneous Mexican hat ring valley with cost *c* = 0.2. The value of *ϕ*_0_ along this ring valley is almost a constant. As cost *c* = 0.24, a new basin emerges at the right corner of the state space shown in Fig 3(C) and 3(G). It denotes the *War* state with larger probability of all defecting strategy *ALLD*. Then at the cost *c* = 0.35, the closed ring valley disappears, only the stable *War* state basin is left shown in Fig 3(D) and 3(H). We notice that the oscillations between *Peace* and *War* can be explained as: when *Peace* state sustains for a long time, the population increases and the resources relatively reduce. In order to survive, the populations fight for the resources, to get better livings. After a war, the populations do not engage in the production, livelihood, and fall into a long-term state of tension. Then the *Peace* state emerges again, the population regrows. The oscillations will thus circulate.

−∇*U* is the negative gradient of the population landscape and −∇*ϕ*_0_ is the negative gradient of the intrinsic landscape. −∇*U* at the top row and −∇*ϕ*_0_ at the bottom row are represented by black arrows. **J**_*ss*_/*P*_*ss*_ is the steady state flux divided by steady state probability and *V* = **J**_*ss*_/*P*_*ss*_)|_*D*→0_ is the intrinsic flux velocity. The flux velocity satisfies **V** ⋅ ∇*ϕ*_0_ = 0 [24]. This shows that the intrinsic flux velocity is perpendicular to the gradient of the non-equilibrium intrinsic potential *ϕ*_0_ in the zero-fluctuation limit [24]. **J**_*ss*_/*P*_*ss*_ at the top row and *V* at the bottom row are represented by purple arrows in Fig 3. We can see that the flux with purple arrows are nearly but not exactly perpendicular to the negative gradient of *U* with the black arrows around the basins or the closed ring valley shown on the top row. The region with higher population potential has some disordered oriented arrows due to the lower probability and limit of the computational accuracy. The flux velocities with purple arrows are exactly perpendicular to the negative gradient of *ϕ*_0_ with black arrows at the bottom row. It is due to **V** ⋅ ∇*ϕ*_0_ = 0. The landscape’s gradient force ∇*U* or the ∇*ϕ*_0_ attracts the system down to the basin or the closed ring valley, while the flux drives the periodical oscillation flow or spiral descent to the basin. It is necessary to characterize this non-equilibrium repeated Prisoner’s Dilemma with both landscape and flux.

**Fig 4:**
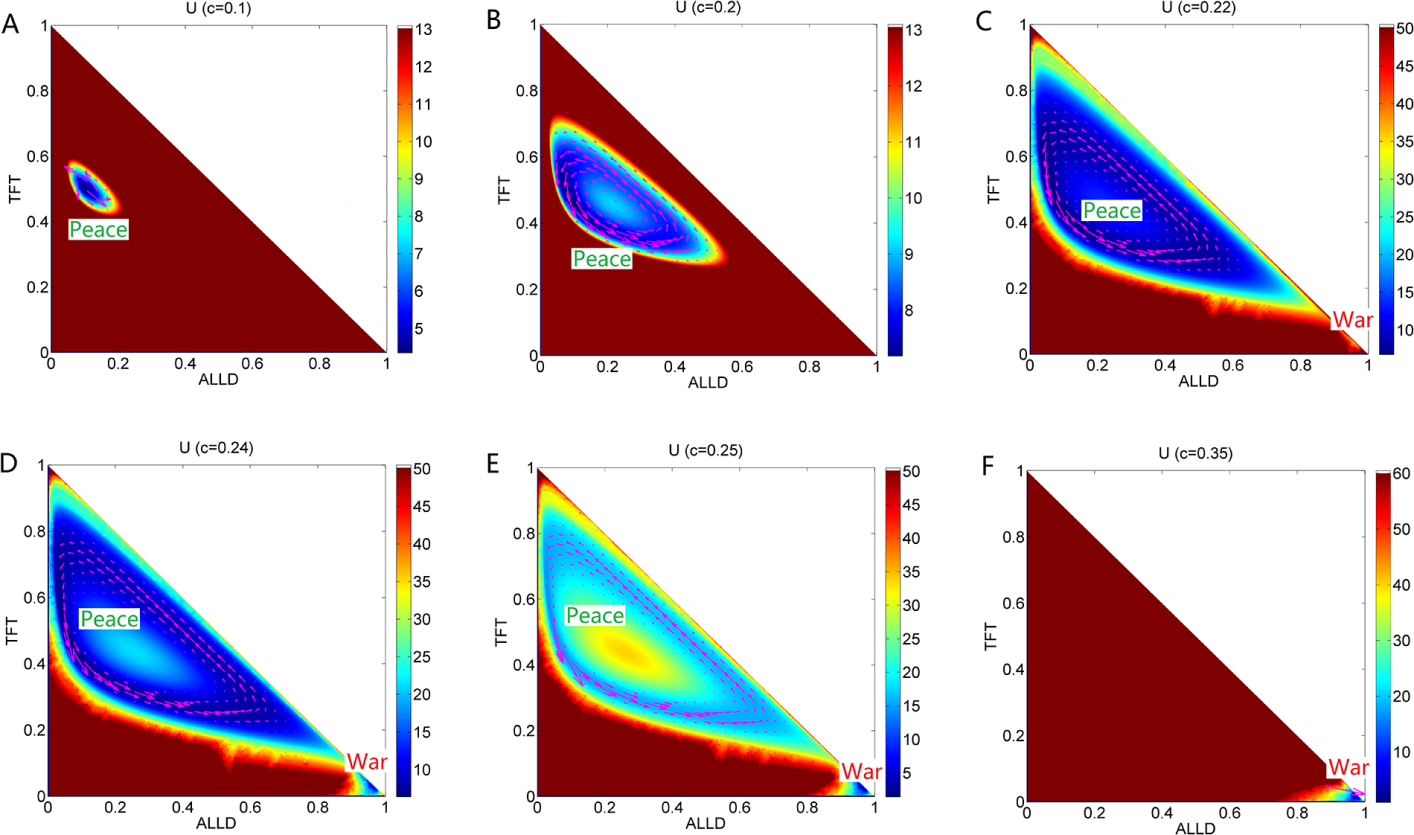
The 2 dimensional population landscape *U* with different parameter *c* and constant parameter *μ* = 0.006. A:*c* = 0.1, B:*c* = 0.2, C:*c* = 0.22, D:*c* = 0.24, E:*c* = 0.25, F:*c* = 0.35. The purple arrows represent the probability flux.

We show the 2 dimensional population landscape *U* for increasing parameter cost *c* with constant *μ* = 0.006 in Fig 4. Fig 4(A) shows that the population landscape *U* has a stable basin near the middle of *TFT* axis with the cost parameter *c* = 0.1. It means that most players choose strategies of *ALLC* and *TFT* when the cost parameter for *TFT* is small. The fluxes represented by the purple arrows rotate anticlockwise around this stable state. This state can be viewed as “Peace” state in peace and war game. As the cost parameter for *TFT* increases to *c* = 0.2, a limit cycle emerges and replaces the stable state in Fig 4(B). The population landscape has a blue ring valley along the deterministic trajectory. More players choose *TFT* and *ALLC* in the *Peace* state. As more players mutate to *ALLC*, a small number of *ALLD* players emerge. This leads to a state with more *ALLD* players. As the *ALLD* players become more, the profit obtained from the game becomes less. Some *ALLD* players convert their strategy to *TFT*. This makes a circle in the state space of strategy probability. Notice that the ring valley is not homogeneous in landscape depth. There is a deeper area on the left side of the limit cycle, which is still close to *TFT* axis. This indicates that the *Peace* state with deeper depth is more stable than other states. The system will stay in *Peace* state much longer than any other state. Fig 4(C) shows the ring valley of the oscillation expands its amplitude in the strategy-frequency space. A stable state *War* emerges at the right corner of the triangle, which is close to *ALLD* → 1. The stable *War* state is the one where most of players choose the *ALLD* strategy. We can see that the limit cycle and the stable state coexist in the strategy-frequency space. It indicates that the system is sometimes in the limit cycle and sometimes in the stable state. The game system can switch between the *Peace* and *War* attractor basins under the fluctuations and the mutations. When the cost for *TFT* increases to *c* = 0.24 and *c* = 0.25 shown in Fig 4(D) and 4(E), the ring valley becomes shallower and shallower while the basin of the stable *War* state becomes deeper and deeper. As *c* increases further, the profits obtained from the *TFT* strategy decreases in the whole game, more players give up *TFT* strategy and choose *ALLD* strategy to earn more, which leads to more stable and deeper *War* state basin. When the cost for *TFT* increases to *c* = 0.35 shown in Fig 4(F), the oscillation ring valley disappears while the *War* state survives and becomes deeper and more stable. Fig 4 also shows the changes in both direction and the scale of each flux at the area with higher probability (purple arrows) when the cost *c* increases. We can see that the fluxes have a anticlockwise rotational nature along the limit cycle. The flux is the driving force for the stable oscillation in game theory.

**Fig 5:**
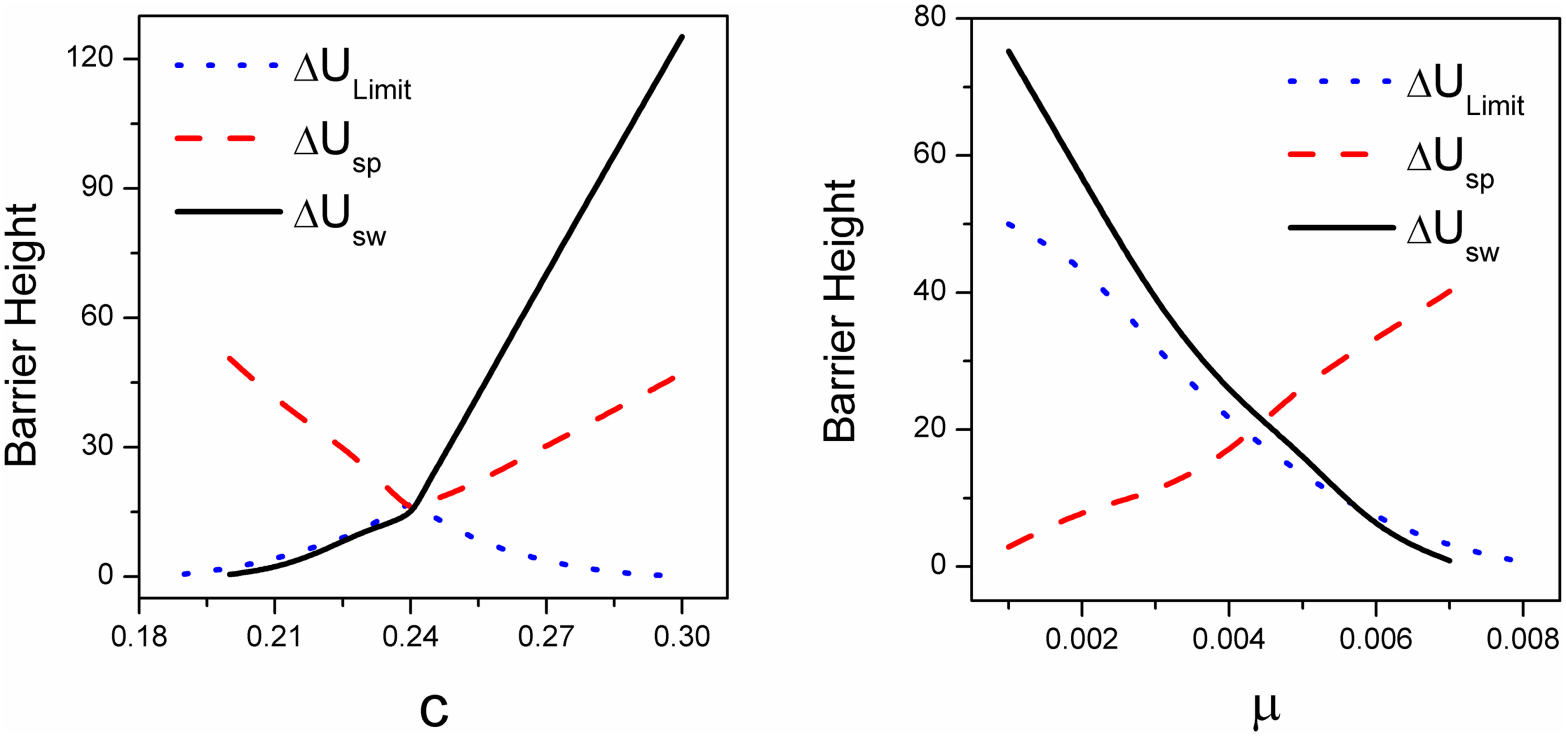
The barrier heights versus parameter *c* and *μ*. A: The barrier heights versus parameter *c* with the parameter *μ* = 0.006. B: The barrier heights versus parameter *μ* with the parameter *c* = 0.22. Here, Δ*U*_*Limit*_ = *U*_*o*_ − *U*_*p*_, Δ*U*_*sp*_ = *U*_*s*_ − *U*_*p*_, Δ*U*_*sw*_ = *U*_*s*_ − *U*_*w*_. *U*_*o*_ is the value of population potential landscape *U* at the maximum point on the center island of the limit cycle. *U*_*s*_ is the value of population potential landscape *U* at the saddle point between the limit cycle valley and the stable state basin *War*. *U*_*p*_ is the minimum value of the population potential landscape *U* along the limit cycle near the *y* axis, which is the *Peace* state. *U*_*w*_ is the minimum value of population potential landscape *U* at the stable state *War*.

We quantify the landscape topography and show barrier heights versus cost *c* with mutation parameter *μ* = 0.006 in Fig 5(A). We first set *U*_*o*_ as the value of population landscape *U* at the maximum point at the center island of the limit cycle. *U*_*s*_ is the value of population landscape *U* at the saddle point between the limit cycle valley and the stable *War* state basin. *U*_*p*_ is the minimum value of population landscape *U* along the limit cycle near *y* axis, which is the *Peace* state. *U*_*w*_ is the minimum value of population landscape *U* at the stable *War* state. We set the barrier height for the oscillation ring valley as Δ*U*_*Limit*_ = *U*_*o*_ − *U*_*p*_, the barrier height between the saddle point and the oscillation as Δ*U*_*sp*_ = *U*_*s*_ − *U*_*p*_ and the barrier height between the saddle point and the *War* stable state as Δ*U*_*sw*_ = *U*_*s*_ − *U*_*w*_. We can see as the cost *c* increases, barrier height Δ*U*_*sw*_ increases, barrier height Δ*U*_*sp*_ decreases first then increases, barrier height Δ*U*_*Limit*_ increases first then decreases. It indicates that the oscillation itself relative to the maximum point in the center of limit cycle becomes more stable first then becomes less stable. It has a turning point during the process of *c* increasing. The *War* state becomes more robust, and the barrier height from oscillation to *War* state Δ*U*_*sp*_ becomes less than that of Δ*U*_*sw*_ with larger cost *c* value. This implies that the *War* attractor state becomes more preferred than that of the oscillation, as the cost increases further.

### Phase diagram, state switching, landscapes and fluxes upon mutations

Fig 2(B) shows the phase diagram for the repeated Prisoner’s Dilemma model with changing mutation parameter *μ* at the constant cost parameter *c* = 0.22. There are four regions in this phase diagram. When the mutation rate *μ* is small, the system has a stable state (can be viewed as *War* state) in Region IV. As *μ* increases, a stable state *War*, an unstable saddle state and the limit cycle coexist in Region III. As the mutation rate *μ* increases further, the stable state and saddle point disappear after the saddle-node bifurcation. There is only limit cycle left in Region II. As the mutation rate *μ* keeps on increasing, the limit cycle becomes a stable *mixed* state (with moderate probability of more than 20% of the players with *ALLD* strategy) in Region I. The *mixed* state is the combination of these three strategies. We can see entropy production rate characterizing the heat dissipation *EPR* shown in Fig 2(D) has a bell shape as the mutation rate *μ* increases when the cost *c* is moderate. This is because that the state of oscillation costs more energy in the strategy probability state space and one stable state costs less energy.

**Fig 6:**
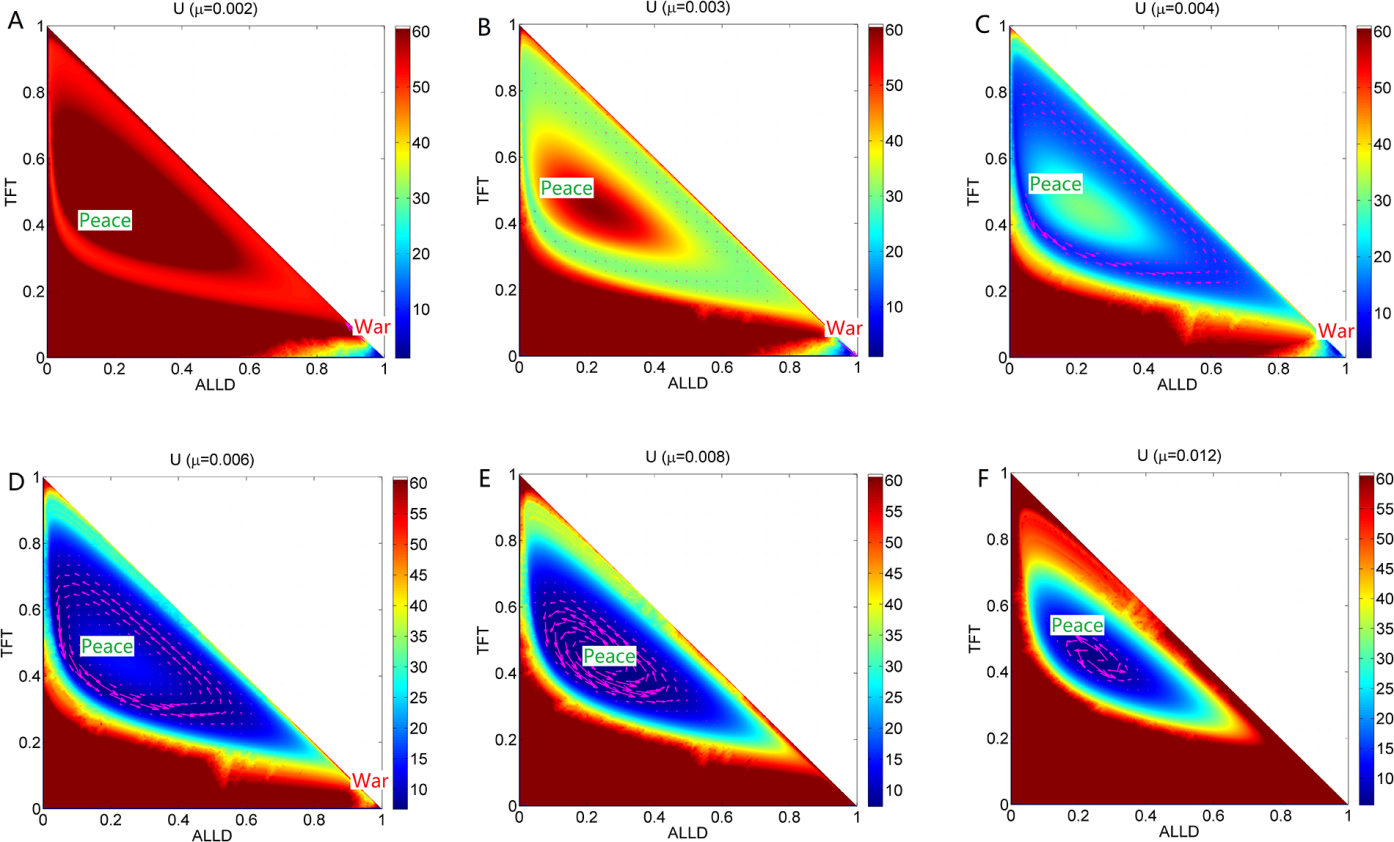
The flux (purple arrows) on the population potential landscape *U* with different parameter *μ* and constant parameter *c* = 0.22. A:*μ* = 0.002, B:*μ* = 0.003, C:*μ* = 0.004, D:*μ* = 0.006, E:*μ* = 0.008, F:*μ* = 0.012.

We can also see this process of transition in Fig 6 which shows that the population landscape *U* and the flux change with the increasing mutation rate *μ* at constant cost *c* = 0.22. Fig 5(B) shows that Δ*U*_*sp*_ increases and Δ*U*_*sw*_, Δ*U*_*Limit*_ decrease as mutation rate *μ* increases. This shows that when mutation rate is small, the limit cycle ring valley is very stable relative to its oscillation center, but has less probability relative to the *War* state since the *War* state is much deeper. It indicates that more players choose strategy *ALLD*, and the players do not like to mutate to the other two strategies. This leads to more stable *War* state. As *μ* increases, the limit cycle ring valley becomes less stable relative to its oscillation center, but becomes more stable relative to the *War* state. Eventually a much more stable state *Peace* emerges. The state *War* becomes shallower and less stable, and finally diminishes as *μ* increases.

### Phase diagram, state switching, landscapes and fluxes upon temptation payoff

**Fig 7:**
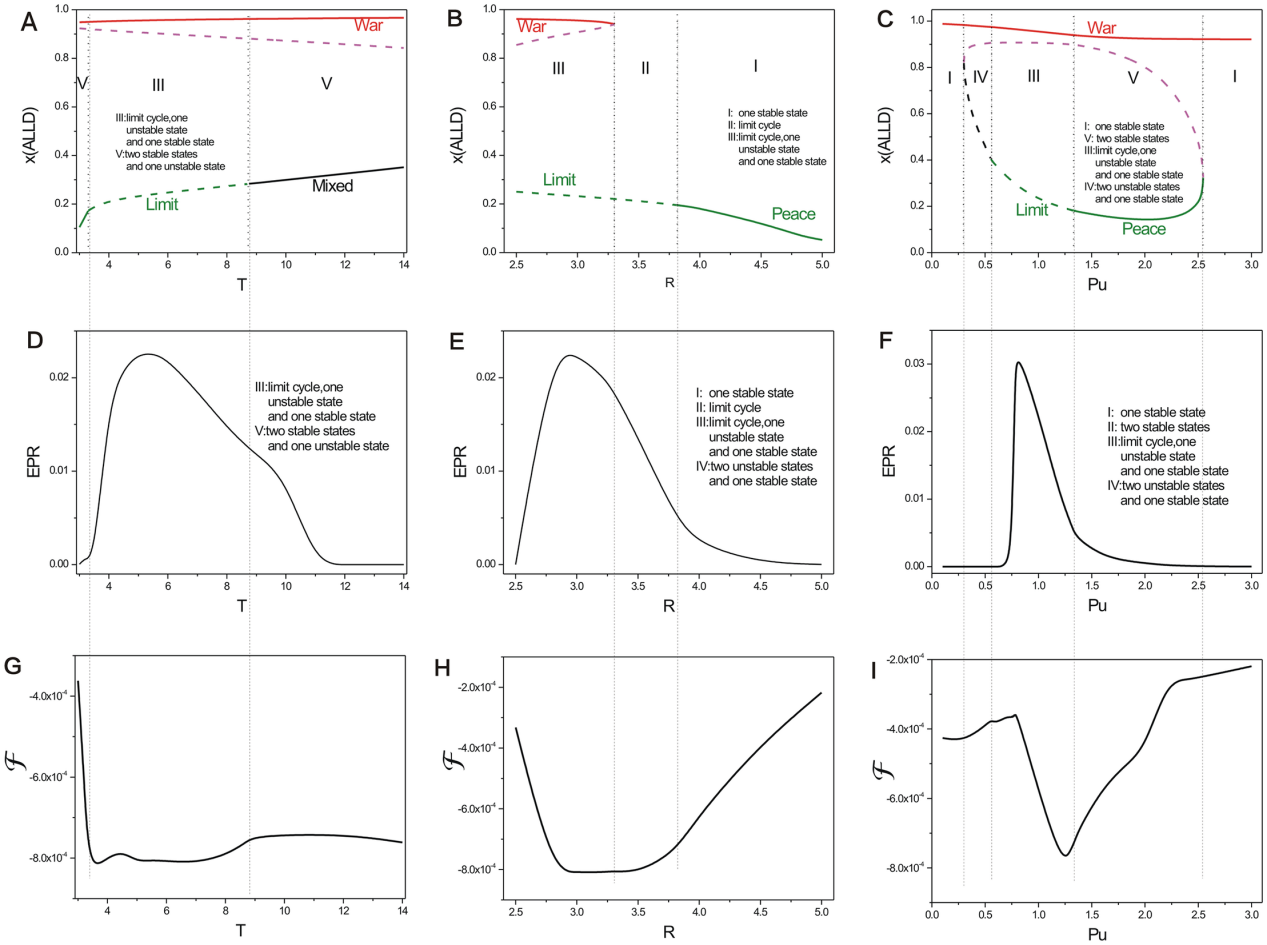
The phase diagrams and the entropy production rate versus parameter *T*, *R*, *Pu*. The phase diagrams for the repeated Prisoner’s Dilemma game model with different parameter *T* (A) *R* (B), *Pu* (C). The entropy production rate versus parameter *T* (D), *R* (E), *Pu* (F). The free energies versus parameter *T* (G), *R* (H), *Pu* (I). The other parameters are *S* = 0, *μ* = 0.006, *c* = 0.22.

**Fig 8:**
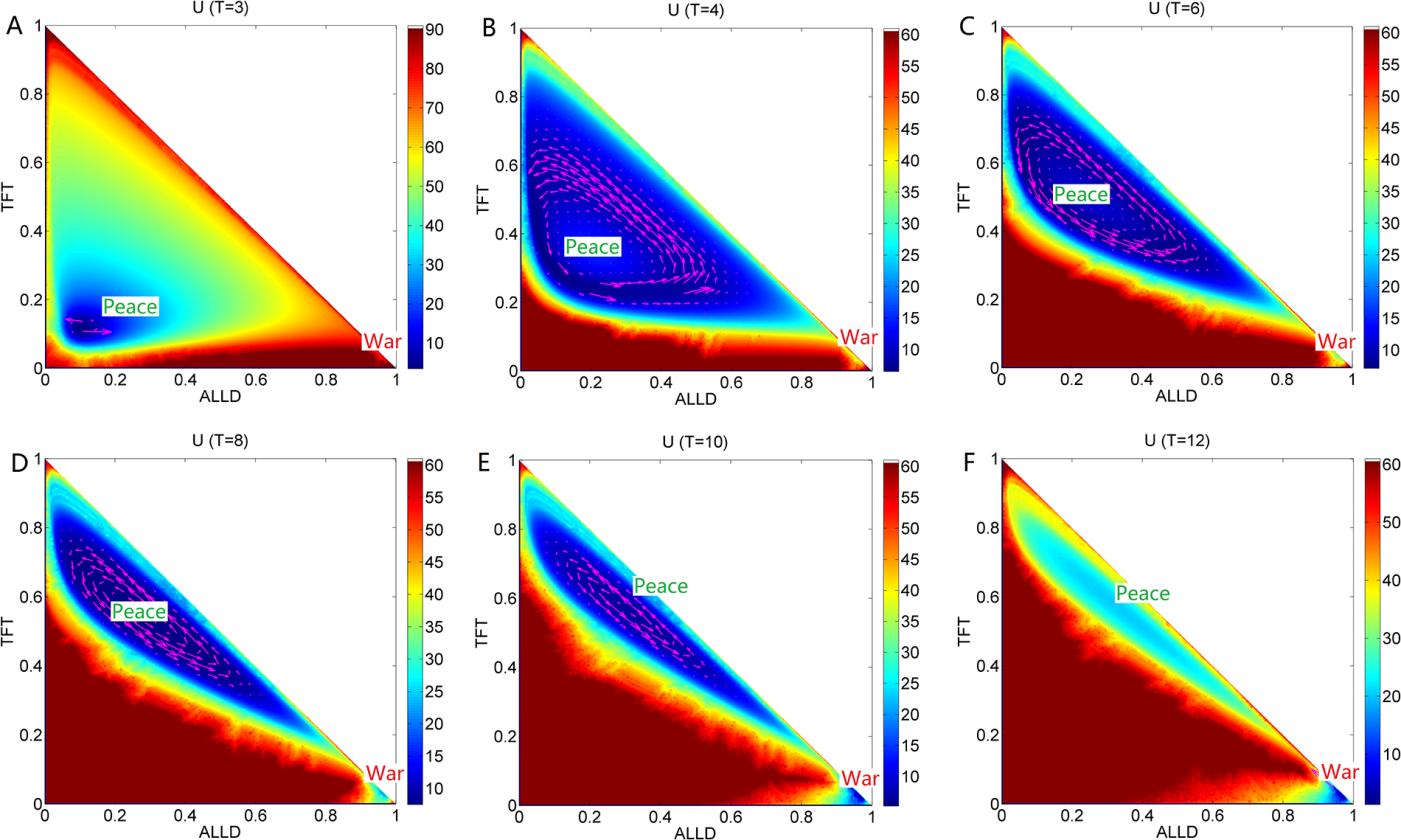
The population landscape *U* and flux for *T*. The population landscape *U* and flux with different parameter *T* at the constant parameters *μ* = 0.006, *c* = 0.22, *R* = 3, *Pu* = 1.0, *S* = 0.0.

**Fig 9:**
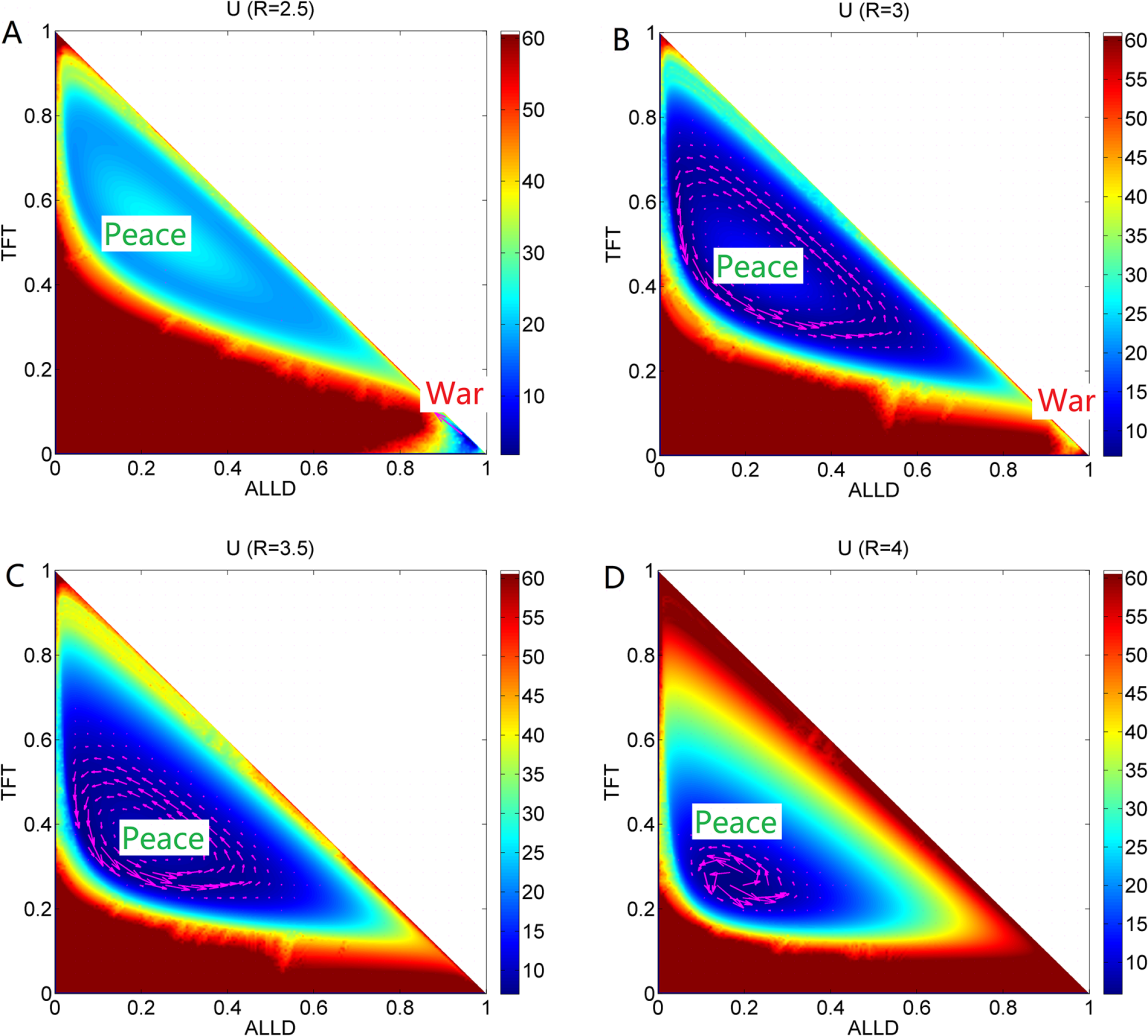
The population landscape *U* and flux for *R*. The population landscape *U* and flux with different parameter *R* at the constant parameters *μ* = 0.006, *c* = 0.22, *T* = 5, *Pu* = 1.0, *S* = 0.0.

**Fig 10:**
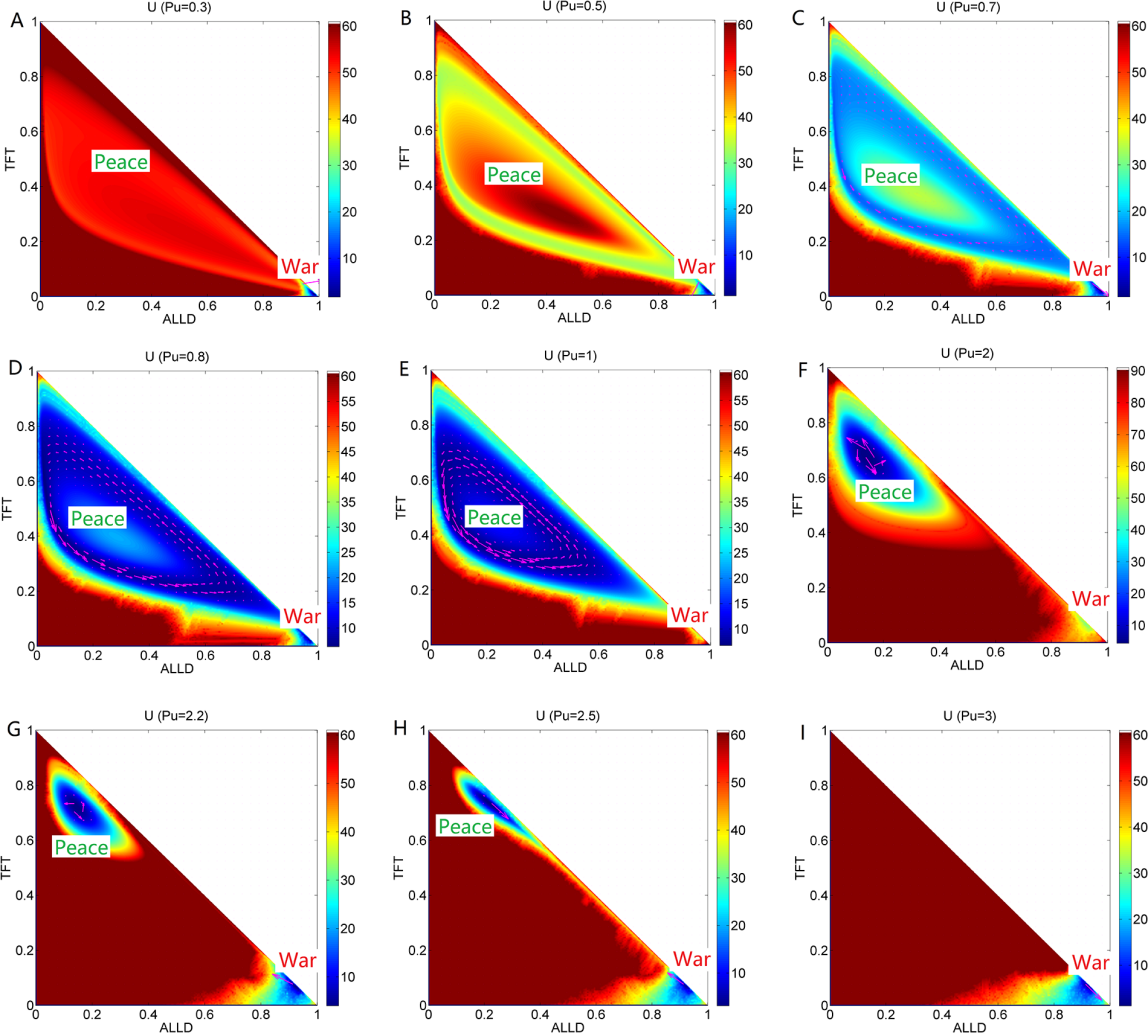
The population landscape *U* and flux for *Pu*. The population landscape *U* and flux with different parameter *Pu* and the constant parameters *μ* = 0.006, *c* = 0.22, *T* = 5, *R* = 3, *S* = 0.0.

Fig 7 shows the phase diagrams, *EPRs*, and free energies for the repeated Prisoner’s Dilemma model versus parameter *T*, *R*, *Pu* in three columns respectively. We put these figures together for comparison and orderliness. Figs 8, 9 and 10 show the population landscape *U* and flux for each parameter *T*, *R*, *Pu* respectively. Firstly, we illustrate Figs 7(A) and 7(D) and 8 for the same parameter *T*. Next we illustrate Figs 7(B), 7(E) and 9 for the same parameter *R*. And then we illustrate Figs 7(C), 7(F) and 10 for the same parameter *Pu*. At last, we focus on discussing the tendencies and the common trends of the free energies with respect to parameters *c*, *μ*, *T*, *R*, *Pu* in Figs 2(E), 2(F) and 7(G), 7(H) and 7(I).

Fig 7(A) shows the phase diagram for the repeated Prisoner’s Dilemma game model with the constant parameters *μ* = 0.006, *c* = 0.22, *R* = 3.0, *Pu* = 1.0, *S* = 0.0 and different parameter *T*. *T* is the temptation payoff earned by the defector while the cooperator gets sucker payoff *S*. There are three regions in this phase diagram. When the temptation *T* is small, the game theory system has two stable states which are denoted as *Peace* and *War* in the peace and war game shown in Region left V. As the temptation *T* increases, a limit cycle emerges with the coexistence of *War* state in Region III. As the temptation *T* increases further, the limit cycle diminishes and a stable *mixed* state emerges, along with a stable state (*War* state) at the right of Region V. We can see *EPR* shown in Fig 7(D) also has a bell shape as the temptation *T* increases. This also implies that limit cycle state cost more energy to maintain its oscillation.

We show that the population landscapes *U* and the flux change with the increasing temptation *T* of payoff matrix (which denotes a defector acquires a temptation payoff *T* when they encounter cooperators) in Fig 8. The game system has two stable states when temptation is very small as *T* = 3. Note that the relationship *T* > *R* should hold in this model. The game system covers a large area of state space with a *Peace* state and a *War* state. We can see that the basin of *Peace* state is very stable and deeper while the *War* state is much shallower and less stable. This illustrates that majority of players choose the cooperation *ALLC* or *TFT* strategy. It leads to a more stable *Peace* state, rather than *War* state with defection strategy *ALLD*, when the temptation is less. Defection can not earn more profits. As temptation increases, the *Peace* state is unstable and becomes a limit cycle state. The limit cycle state adopts the mixed strategy *TFT* and *ALLD*. The limit cycle valley representing the *Peace* state becomes shallower while the *War* state becomes more stable shown in Fig 8(B), 8(C) and 8(D). When the temptation for this game increases even further, more and more players choose defection strategy *ALLD* to earn more profits rather than the strategy *ALLC*. It reflects that more temptation from the defection strategy can lead to more stable *War* state.

### Phase diagram, state switching, landscapes and fluxes upon cooperation reward

Fig 7(B) shows the phase diagram for the repeated Prisoner’s Dilemma game model at the constant parameter *μ* = 0.006, *c* = 0.22, *T* = 5.0, *Pu* = 1.0, *S* = 0.0 and different parameter *R*. The element *R* of payoff matrix denotes the reward that cooperators will acquire from the mutual aid when the cooperators encounter. The value of reward *R* should satisfy *R* > (*T* + *S*)/2. If this relationship is not satisfied, the agreement of alternate cooperation and defection will earn more payoff than that of pure cooperation in a repeated Prisoner’s Dilemma game [6]. There are three regions in this phase diagram. When the reward *R* is small, a limit cycle and the stable *War* state coexist in Region III. As the reward *R* increases, the *War* state disappears after the saddle-node bifurcation and the limit cycle is left in Region II. As the reward *R* increases further, the limit cycle diminishes and a stable *Peace* state emerges in Region I. Fig 7(E) also shows *EPR* versus reward *R*, which implies that the limit cycle state costs more energy than that of one stable state.

Fig 9 shows the population landscape *U* with increasing cooperation *R*. The system has one deep stable *War* basin state and a shallower limit cycle ring valley for small parameter *R* = 2.5, since the reward for cooperation strategy is small. The majority players choose the defection strategy leading to the *War* state shown in Fig 9(A). When the reward increases to *R* = 3, a limit cycle valley becomes deeper and stable while the *War* state becomes shallower and less stable as shown in Fig 9(B). It shows that more and more players prefer the strategy *ALLC* rather than strategy *ALLD* since more reward can be obtained from the cooperation. As reward *R* increases further, the limit cycle valley becomes deeper, and the *War* state vanishes shown in Fig 9(C). As reward *R* increases even further, the limit cycle ring valley shrinks into a stable *Peace* state shown in Fig 9(D). The majority players choose cooperation strategy. It is because more reward from the cooperation strategy can lead to more stable *Peace* state. It turns out that more reward from cooperation leads to *Peace* with win-win outcome.

### Phase diagram, state switching, landscapes and fluxes upon defector punishment

Fig 7(C) shows the phase diagram for the repeated Prisoner’s Dilemma model at the constant parameter *μ* = 0.006, *c* = 0.22, *T* = 5.0, *R* = 3.0, *S* = 0.0 and different parameter *Pu*. The element *Pu* of payoff matrix denotes that the defectors obtain a punishment *Pu* when the defectors encounters. The value of punishment *Pu* should satisfy *R* > *Pu* > *S* [6]. There are five regions in this phase diagram. When the punishment *Pu* is small, only the stable *War* state exists in Region I on the left. As the punishment *Pu* increases, two unstable states emerge after the saddle-node bifurcation in Region IV. As the punishment *Pu* increases further, a limit cycle emerges and coexists with the stable state *War* in Region III; as the punishment *Pu* increases even further, the limit cycle diminishes and a stable *Peace* state coexists with a stable *War* state in Region V. As the punishment *Pu* approaches to 3, the stable *Peace* state diminishes after the saddle-node bifurcation, and the stable *War* state is left in Region I on the right. Fig 7(F) also shows *EPR* versus punishment *Pu*, which shows that the system with limit cycle dissipates more energy.

Fig 10 shows the population landscape *U* with increasing element *Pu* of payoff matrix. When the punishment *Pu* is small representing that the defector can earn less from the game and almost all players choose strategy *ALLD* shown in Fig 10(A). *ALLD* is the only strict Nash solution and the only evolutionary stable strategy. And when the punishment (*Pu* = 0.3) is small, the value of the first element in the left column of the payoff matrix for *ALLD* is *Pu* while the second of that for *TFT* is *Pu* − *c* = 0.08. Thus the fitness for *ALLD* is much higher than that of *TFT*, and that of *ALLC* (The sucker’s payoff *S* = 0). As punishment *Pu* increases, more and more players give up *ALLD* since the profits of *TFT* players are catching up with that of *ALLD* players when they both encounter the *ALLD* players. As the punishment *Pu* increases to *Pu* = 0.5, a shallow limit cycle valley emerges accompanied with the stable *War* state shown in Fig 10(B). When the punishment *Pu* increases further, the limit cycle valley shrinks its size but becomes deeper and more stable while the *War* state becomes shallower and less stable. This is shown in Fig 10(C), 10(D) and 10(E). Then the limit cycle ring valley shrinks to a stable *Peace* state where more players choose *TFT* strategy as shown in Fig 10(F), 10(G) and 10(H). This shows that as the punishment *Pu* is increased further, the *Peace* state becomes more stable first and then loses its stability while the *War* state becomes more stable. It demonstrates that the punishment *Pu* has an optimal value to lead to a more stable *Peace* state. At last, when the value of the punishment *Pu* approaches to the value of reward *R*, the stable *Peace* state diminishes and the stable *War* state is left in Fig 10(I). This shows that when the punishment *Pu* and the reward *R* are almost the same, *ALLD* is dominant than *TFT* since *ALLD* is the only strict Nash equilibrium solution and the evolutionary stable state. These results show that the strategy *ALLD* can be a dominant strategy and has an advantage for selection.

We explored the free energy versus the parameters of the repeated Prisoner’s Dilemma game model in Fig 2(E) (the free energy versus cost *c*), Fig 2(F) (the free energy versus the mutation rate *μ*), Fig 7(G) (the free energy versus temptation *T*), Fig 7(H) (the free energy versus reward *R*), Fig 7(I) (the free energy versus punishment *Pu*). We can see this five free energy profiles have some similarity that each has the opposite tendency with that of the corresponding *EPR*. These free energies link to the different phases and phase transitions. The first derivative of the free energies are discontinuous at the transition points from a stable state to a limit cycle oscillation state and vice versa [24]. This implies that non-equilibrium thermodynamic phase transition has certain similarities as that of the equilibrium thermodynamic phase transition. Free energy profiles can manifest the phase transitions and can be used to explore the global stability and robustness of the game system.

### Kinetic speed and optimal paths of switching between the two stable states

We also explored the kinetic optimal paths of the repeated Prisoner’s Dilemma game model. We can obtain the relative probabilities of each path by the quantification of the path weights with path integrals. Path integral weights can be calculated by the action, which is analogous to the classical mechanical systems. The dominant optimal paths with the largest weights can be viewed as the major pathways. The path integral for the probability of (**x**_**final**_, *t*) starting at initial condition (**x**_**initial**_, 0) is given as [21, 24]:

$$\begin{array}{ccc} P(x_{\text{final}},t,x_{\text{initial}},0) & = & \int DxExp[-\int dt(\frac{1}{2}\nabla \cdot F\left(x\right)\\ & & +\frac{1}{4}\left(dx/dt-F\left(x\right)\right)\cdot \frac{1}{D(x)}\cdot \left(dx/dt-F\left(x\right)\right))]\\ & = & \int DxExp[-A(x)] \end{array}$$ (12)

The above probability describes the chance of starting at the state **x**_**initial**_ at initial time and ending at the state **x**_**final**_ at the final time. The probability is the result of the sum of the weights from all possible paths. *A*(**x**) is the action for each path. Each weight is exponentially related to the action which has two contributions, one from the stochastic equation of motion for the dynamics and the other is from the variable transformation from the stochastic force to the system variable. Not all the paths contribute equally to the weight. Due to the exponential nature, the optimal path is exponentially larger in weight than the suboptimal ones. Therefore, we can identify the optimal path with the most probable weight.

**Fig 11:**
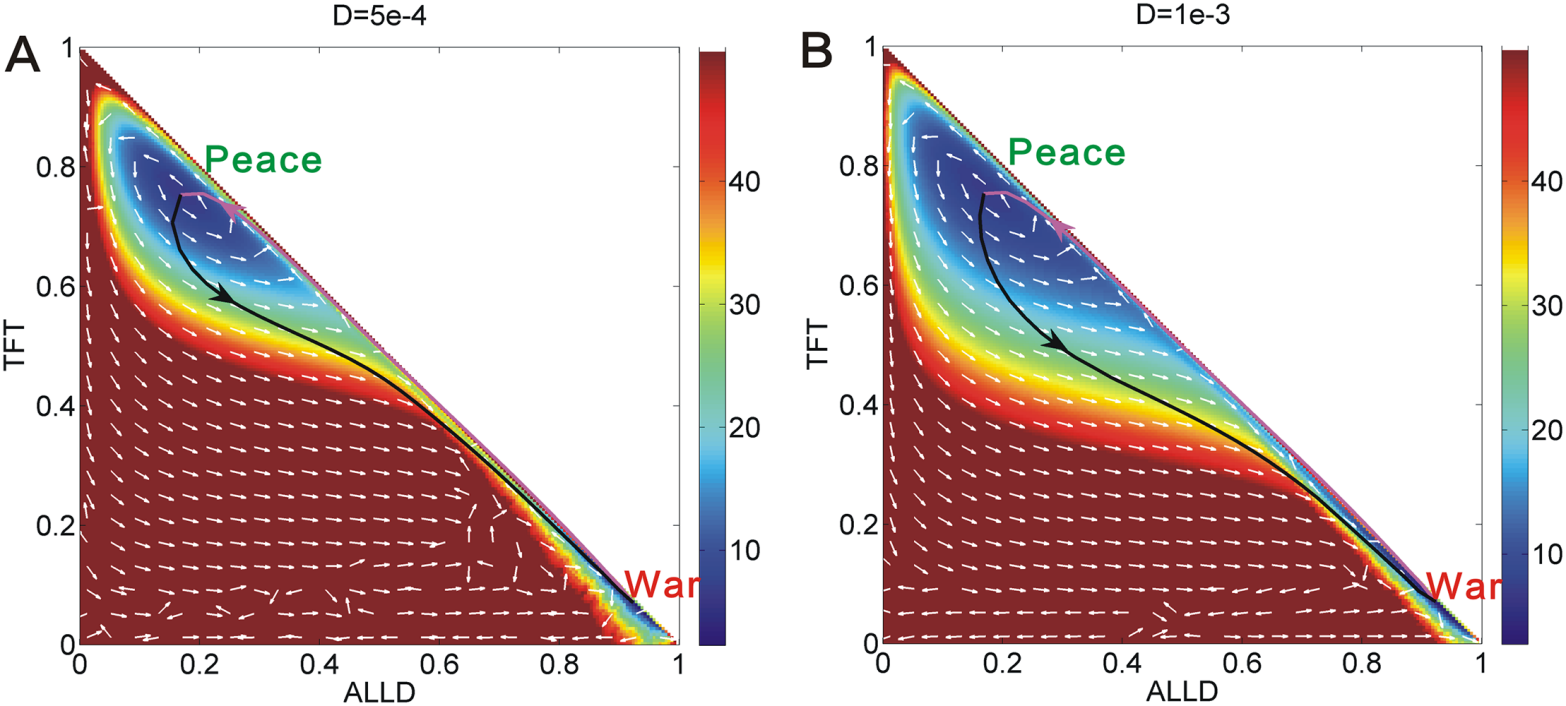
The pathways on the population potential landscape *U* with different diffusion coefficient *D* at *μ* = 0.006, *c* = 0.22, *T* = 5, *R* = 3, *Pu* = 2.4, *S* = 0.0. The purple lines represent the optimal paths from the *War* state to *Peace* state. The black lines represent the optimal paths from the *Peace* state to *War* state. The white arrows represent the steady state probability fluxes.

We studied the repeated Prisoner’s Dilemma game model with the parameters *μ* = 0.006, *c* = 0.22, *T* = 5, *R* = 3, *Pu* = 2.4, *S* = 0.0, which has two stable state *Peace* and *War*. Fig 11 shows the optimal paths on the population landscape *U* with different diffusion coefficient *D*. We can see there are two stable states: *War* and *Peace* on the population landscapes. The purple lines represent the optimal paths from the *War* state to *Peace* state. The black lines represent the optimal paths from the *Peace* state to *War* state. The white arrows represent the steady state probability fluxes which guide the optimal paths apart from the steepest descent path from the landscape. Therefore, the optimal path from *War* state to *Peace* state and the optimal path from *Peace* state to *War* state are apart from each other. Under more fluctuations (bigger diffusion coefficient *D* shown in Fig 11(B)), the two optimal paths are further apart from each other due to larger probability fluxes than those in Fig 11(A). We can see the purple lines and black lines are irreversible in both two sub figures. The optimal paths are deviated from the naively expected steepest descent paths based on the potential landscape. These lines are apart from each other due to the non-zero flux. We can clearly see the fluxes have spiral shapes which show the dynamic feature of non-equilibrium system.

**Fig 12:**
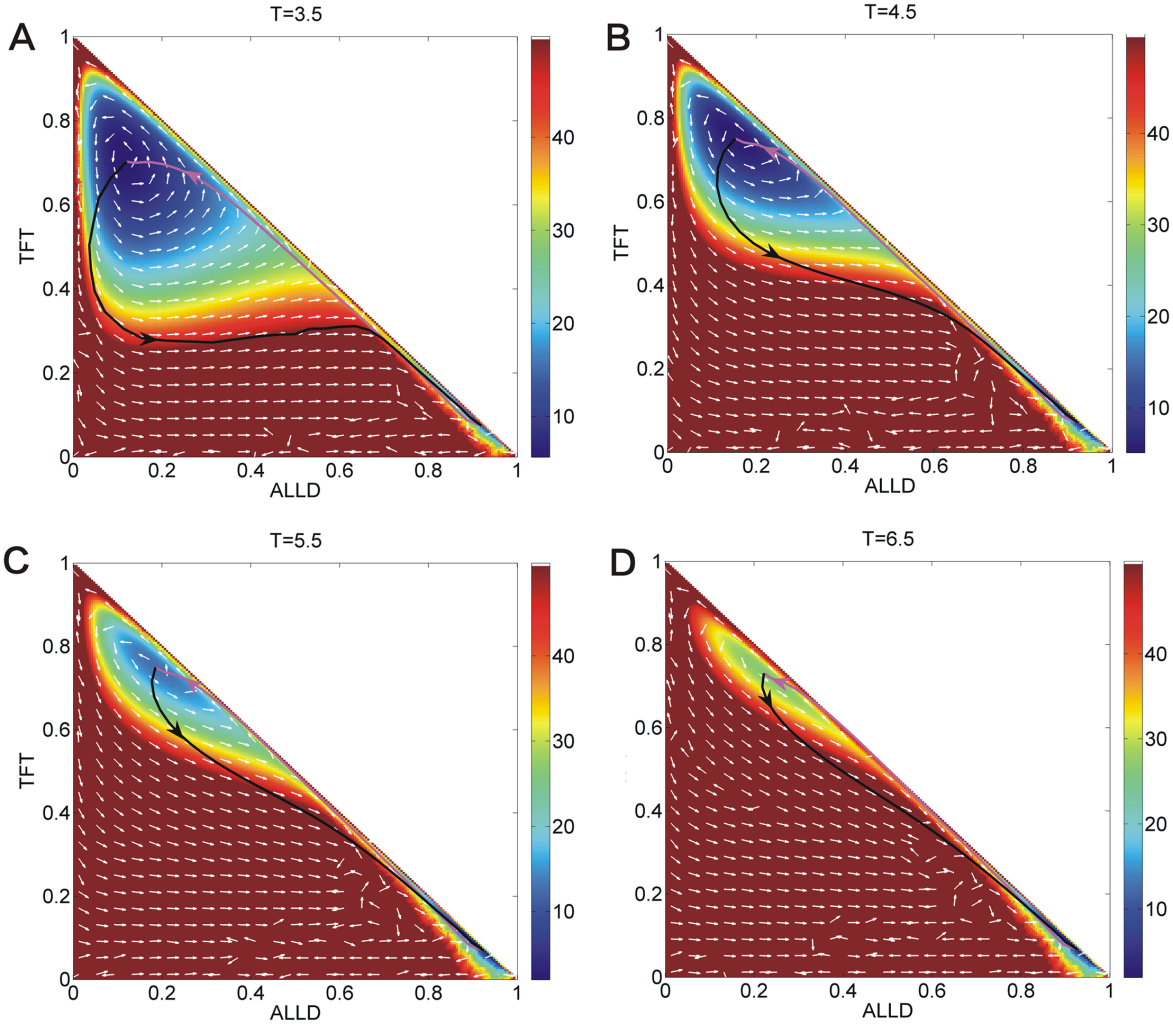
The pathways on the population landscape *U* with different parameter *T* at the constant parameters *μ* = 0.006, *c* = 0.22, *R* = 3, *Pu* = 2.4, *S* = 0.0. The purple lines represent the optimal paths from the *War* state to *Peace* state. The black lines represent the optimal paths from the *Peace* state to *War* state. The white arrows represent the steady state probability fluxes.

**Fig 13:**
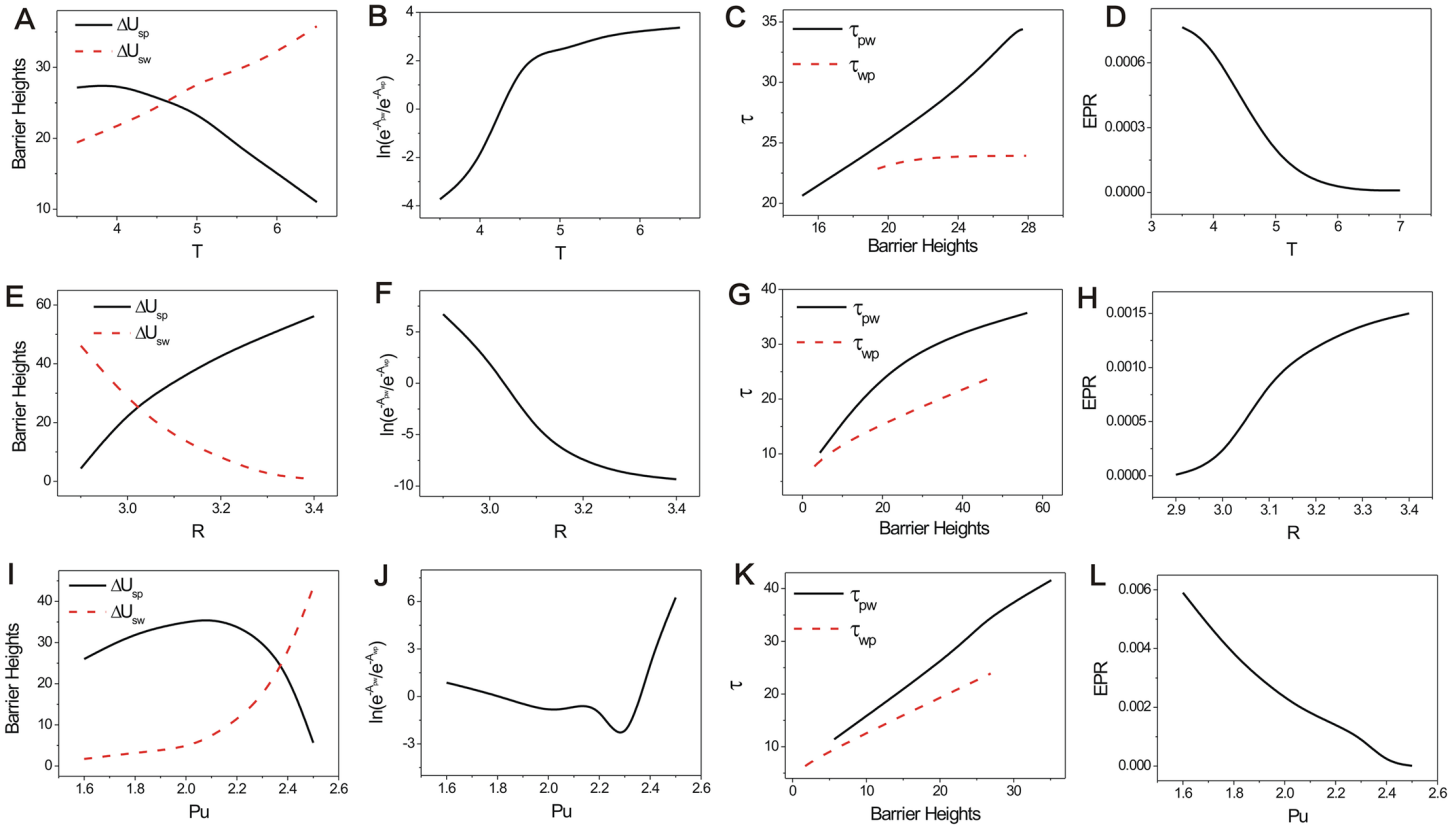
The barrier heights, the logarithm of the ratio of the probability of dominant optimal path, the logarithm of the escape time *MFPT*, entropy production rate versus *T*, *R*, *Pu*. A: the barrier heights versus *T*, B: the logarithm of the ratio of the probability of dominant optimal path from *Peace* state to *War* state and the probability of dominant optimal path from *War* state to *Peace* state versus *T*, C: the logarithm of the escape time *MFPT* versus *T*, D: entropy production rate versus *T*, E: the barrier heights versus *R*, F: the logarithm of the ratio of the probability of dominant optimal path from *Peace* state to *War* state and the probability of dominant optimal path from *War* state to *Peace* state versus *R*, G: the logarithm of the escape time *MFPT* versus *R*, H: entropy production rate versus *R*, I: the barrier heights versus *Pu*, J: the logarithm of the ratio of the probability of dominant optimal path from *Peace* state to *War* state and the probability of the dominant optimal path from *War* state to *Peace* state versus *Pu*, K: the logarithm of the escape time *MFPT* versus *Pu*, L: entropy production rate versus *Pu*.

We show the population landscape *U* and the optimal paths with different parameter temptation *T* at the constant parameters *μ* = 0.006, *c* = 0.22, *R* = 3, *Pu* = 2.4, *S* = 0.0, *D* = 5 × 10^−4^ in Fig 12. The purple lines represent the optimal paths from the *War* state to *Peace* state. The black lines represent the optimal paths from the *Peace* state to *War* state. The white arrows represent the steady state probability fluxes. The optimal paths are deviated from the naively expected steepest descent optimal paths based on the potential landscape, and they are irreversible. The two optimal paths become more closer from each other as *T* increases. Fig 13(A) shows the barrier heights versus temptation *T*. We set Δ*U*_*sp*_ = *U*_*saddle*_ − *U*_*Peace*_ as the barrier height between state *Peace* and the saddle point and Δ*U*_*sw*_ = *U*_*saddle*_ − *U*_*War*_ as the barrier height between state *War* and the saddle point, where *U*_*saddle*_ represents the value of landscape *U* at the saddle point between state *Peace* and *War*, *U*_*Peace*_ represents the minimum value of landscape *U* at state *Peace*, *U*_*War*_ represents the minimum value of landscape *U* at state *War*. We can see that the barrier height Δ*U*_*sw*_ increases while the barrier height Δ*U*_*sp*_ decreases. It shows the *Peace* state loses its stability as the *War* state becomes more stable as the temptation *T* increases. It denotes that the temptation guides more players to choose strategy *ALLD*. The path weight represents the probability of each route. The path probability can be obtained by action *A*(*x*) between Peace and War. We labeled *A*_*wp*_ as the action of the dominant optimal path from *War* state to *Peace* state, and *A*_*pw*_ as the action of the dominant optimal path from *Peace* state to *War* state. Fig 13(B) showed the logarithm of the probability of the *Peace* to *War* optimal path divided that of *War* to *Peace* optimal path increases as the temptation *T* increases. This shows that the optimal path from *Peace* state to *War* state has more weight or chance than that of *War* state to *Peace* state due to the losing stability of *Peace* state which lowers the barrier from *Peace* state to *War* state. This originates from the increasing temptation which guide more players to choose strategy *ALLD*.

**Fig 14:**
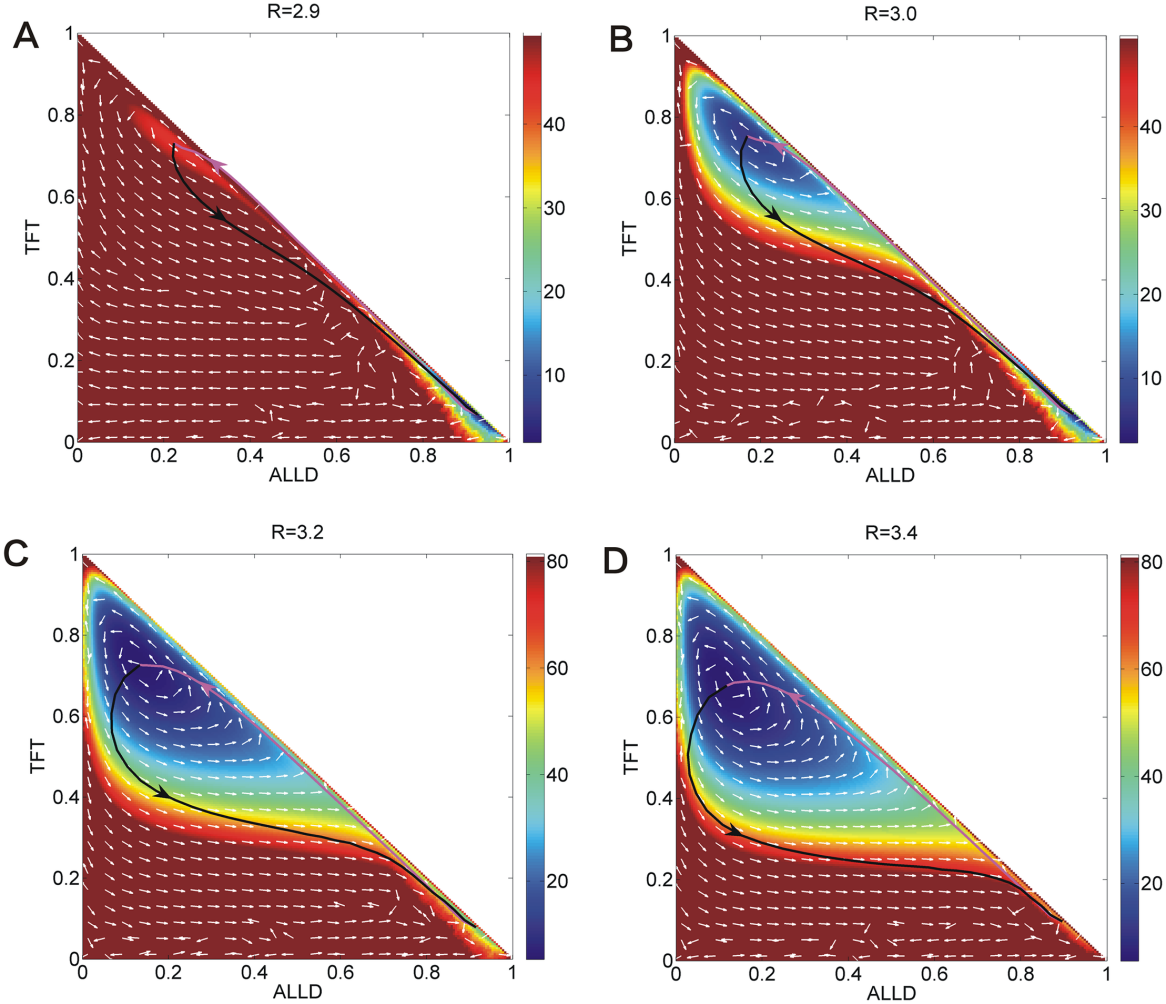
The pathways on the population landscape *U* with different parameter *R* at the constant parameters *μ* = 0.006, *c* = 0.22, *T* = 5, *Pu* = 2.4, *S* = 0.0. The purple lines represent the optimal paths from the *War* state to *Peace* state. The black lines represent the optimal paths from the *Peace* state to *War* state. The white arrows represent the steady state probability fluxes.

We show the population landscape *U* and the optimal paths with different parameter reward *R* at the constant parameters *μ* = 0.006, *c* = 0.22, *T* = 5, *Pu* = 2.4, *S* = 0.0 in Fig 14. The optimal paths do not follow the steepest descent paths based on the potential landscape. The two optimal paths depart far away from each other as *R* increases. Fig 13(E) shows the barrier height Δ*U*_*sw*_ decreases while the barrier height Δ*U*_*sp*_ increases as reward *R* increases. Fig 13(F) showed the logarithm probability of *Peace* to *War* optimal path divided that of *War* to *Peace* optimal path decreases as reward *R* decreases. This shows that as the reward increases, more and more players choose strategy *ALLC* which makes the *Peace* state more stable and the War state less stable. Thus, the optimal path from state *War* to state *Peace* has more probability or weight (chance) than the opposite path.

**Fig 15:**
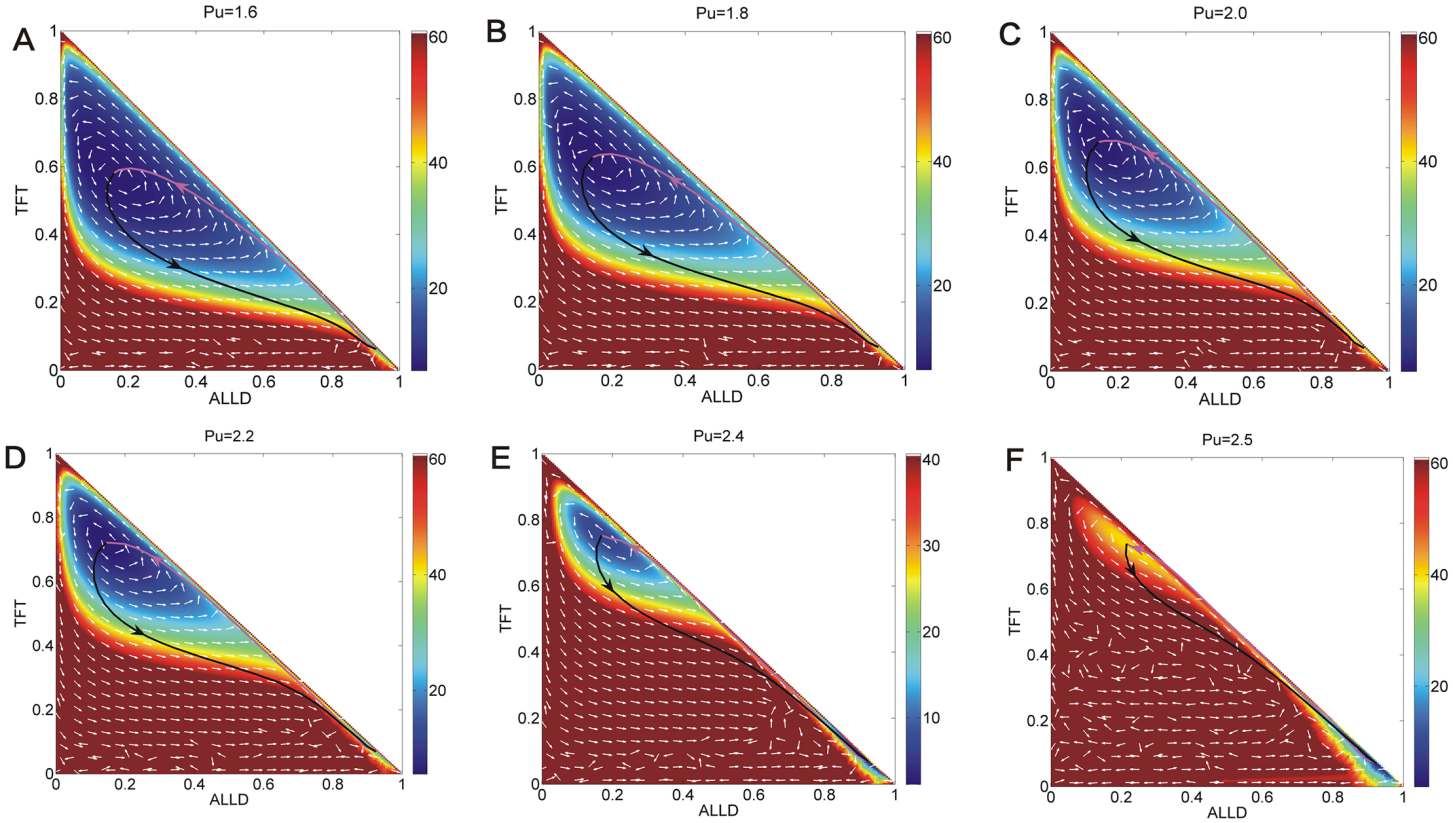
The population landscape *U* with different parameter *Pu* at the constant parameters *μ* = 0.006, *c* = 0.22, *T* = 5, *R* = 3, *S* = 0.0. The purple lines represent the optimal paths from the *War* state to *Peace* state. The black lines represent the optimal paths from the *Peace* state to *War* state. The white arrows represent the steady state probability fluxes.

We show the population landscape *U* and the optimal paths with different parameter punishment *Pu* at the constant parameters *μ* = 0.006, *c* = 0.22, *T* = 5, *R* = 3, *S* = 0.0 in Fig 15. The two optimal paths do not follow the steepest descent optimal paths based on the potential landscape and come closer from each other as *Pu* increases. Fig 13(I) shows that the barrier height Δ*U*_*sw*_ increases while the barrier height Δ*U*_*sp*_ increases first and then decreases as punishment *Pu* increases. Fig 13(J) showed that the logarithm of *Peace* state to *War* state optimal path probability divided that of *War* state to *Peace* state optimal path decreases first and then increases as punishment *Pu* increases. This shows that as the punishment increases, more players choose strategy *ALLC* and *ALLD* which makes the *Peace* state and War state both become more stable. As the punishment grows bigger, the *War* state becomes more stable than that of the *Peace* state. This shows that the punishment *Pu* has an optimal value to make the *Peace* state stable.

We explored the escape time for the evolutionary game system. We used the following equation to determine the escape time *τ*: **F** ⋅ ∇*τ* + *D* ⋅ ∇^2^*τ* = −1 [28]. The escape time can be viewed as the average time spent for a system from one state to another [24]. We can set the mean first passage time (MFPT) *τ*_*pw*_ representing the MFPT from *Peace* state to *War* state and *τ*_*wp*_ representing the MFPT from *War* state to *Peace* state.

Fig 13(C), 13(G) and 13(K) shows that the logarithm of the MFPT increases as their corresponding barrier heights increase corresponding to Fig 13(A), 13(E) and 13(I). The logarithm of the MFPT and barrier heights have the positive correlation as: *τ* *exp*(Δ*Ba*). The average kinetic speeds of the state switching along the corresponding optimal paths can be measured by the 1/*τ*. As the barrier height becomes higher, the escape time becomes longer and the kinetic speed becomes slower. Therefore, the state is more stable with higher barrier height. It is more difficult to switch from one basin of attraction to another with higher barriers between two stable states. It takes more time for switching and the kinetic speed is slower. The *MFPT*, kinetic speed and barrier height can provide the measurements for quantifying the stability of game theory systems. They can also give more quantitative information about the dynamics of a game theory system. This can help to uncover the underlying mechanisms of the state transitions in the game theory systems.

Fig 13(D), 13(H) and 13(L) shows the entropy production rate versus parameters *T*, *R*, *Pu* among the phase range of the two stable states. *EPR* decreased as temptation *T* increases, while *Peace* state loses its stability and *War* state becomes more stable shown in Fig 13(D). *EPR* increased as reward *R* increases, while *War* state loses its stability and *Peace* state becomes more stable shown in Fig 13(H). *EPR* decreased as punishment *Pu* increases, while *Peace* state loses its stability and *War* state becomes more stable shown in Fig 13(L). We can clearly see that the system with dominant *Peace* state will cost more dissipation quantified by *EPR* than that with dominant *War* state. In order to illustrate this phenomenon, we should explore the linear stability of the repeated Prisoners’ Dilemma game model. The eigenvalues of *Peace* state have negative real parts and two opposite imaginary parts. This indicates that *Peace* state is a stable focus which oscillates and spirals to its destiny. Stable focus can switch to an unstable focus which represents a limit cycle. The eigenvalues of *War* state have negative real parts and no imaginary parts. This indicates that *War* state is a stable node. Since the stable focus costs more dissipation. This shows that keeping the peace will consume more energy.

## Discussion and conclusion

Global stability and the underlying mechanism of the dynamics are crucial for understanding the nature of the game theory. Foster and Young presented the analysis of stochastic perturbation of evolutionary game dynamics, defining the stability for a stochastic dynamics [7, 9]. It is viewed as a way of capturing the long-run stability of the stochastic evolutionary game dynamics rather than the evolutionary stable strategy and the Nash equilibrium [7, 9]. They also introduced the idea of a potential function which can be used to compute the stochastically stable set. However, their method can only obtain the potential function in one dimension often in equilibrium. In reality, the game systems are often more complex and in high dimensions. The evolutionary game dynamics are also in general of non-equilibrium. It is difficult to obtain the analytical potential functions to capture the stability of the evolutionary game systems in higher dimensions, since a pure gradient of potential landscape cannot be directly obtained for general evolutionary game dynamics.

Many researchers tried to explore the stability of the evolutionary game systems [6, 7, 9, 17]. Some chose the simulations of the trajectories under fluctuations [7, 17]. The stability of the repeated Prisoner’s Dilemma game can be quantified by the Lyapunov function. But the Lyapunov function cannot be found easily. In this work we developed the landscape and flux theory for quantifying the population and intrinsic landscape of the game theory dynamics. The intrinsic landscape *ϕ*_0_ has a Lyapunov feature which can be used to explore the global stability of the game theory systems. We obtained the numerical Lyapunov function *ϕ*_0_ by solving the Hamilton-Jacobi equation and the population potential landscape *U* from the Fokker-Plank diffusion equation. Thus we can explore the global stability of the game theory system by the intrinsic landscape *ϕ*_0_ and the population potential landscape *U*. The repeated Prisoner’s Dilemma game system is a non-equilibrium system. The underlying non-equilibrium driving dynamics of the game system is determined by both the force from the gradient of the landscape and the force from the steady state probability flux which breaks the detailed balance. This provides a new view to explore the game theory dynamics. In conventional evolutionary game dynamics, the flux is not considered. Here we point out that both the landscape and flux are necessary to study the evolutionary game dynamics.

The barrier height and the entropy production rate can be used to quantify the global stability of the non-equilibrium repeated Prisoner’s Dilemma game. The irreversible optimal paths can be evaluated by the path integral method. The optimal paths are not along the gradient of the landscape due to the non-zero flux. We also quantified the mean first passage time which can measure the kinetic speed of the dynamics of switching from one state to another.

We have found that when the cost for *TFT* which reduces the values of the element of *TFT* in payoff matrix is small, and thus the values of payoff elements for *TFT* are large, the game system approaches to *Peace* state easily. As the cost *c* increases further, the game system will go to the *War* state since the profits from the *TFT* strategy is much less. We have also found that when *c* is small, high mutation rate will lead to the *Peace* state to be far from *War* state.

When *c* is moderate, high mutation rate will lead to a mixed strategy state which has almost the same probability of these three strategies. This leads the game system far from *War* state. However, as the cost *c* is larger, the system will fall into *War* state either with low mutation rate or high mutation rate.

We have also found that moderate intensity of punishment for defection strategy (moderate value of parameter *Pu*) decreases the stability of *War* state. More reward for cooperation strategy (high value of parameter *R*) prefers the *Peace* state. More temptation for the defector from the cooperator will prefer *War* state to earn more profits using defection strategy. Thus choosing a moderate intensity of punishment for defection strategy and increasing the intensity of reward for cooperation strategy will avoid the lasting *War* state, and favor the long lasting *Peace* state.

We provided a path integral method to identify and quantify the optimal paths between each two stable state. The optimal paths between *Peace* states and *War* state are irreversible due to the non-zero flux which is the characteristic for non-equilibrium system. The probability of each optimal path can also give us the information about stability. More stable state has less probability of the corresponding path to escape from its attraction. More time will be spent to escape from more stable state with higher barrier heights as shown from the behavior of *MFPT*. Thus the speeds of swithing between stable states become slower. We have also shown that the game system with dominant stable *Peace* state has more *EPR*. This shows that keeping peace will cost more energy.

Our method can provide a way to identify and quantify the optimal paths (the process) and the kinetics (speed) with their corresponding barrier heights (global landscape topography) for game theory systems. Quantitative study as this will help us to find ways to elongate peace and prevent war.

Since the stochastic game theory dynamics is more difficult to explore analytically, we developed a potential-flux framework to explore and quantify the stochastic game theory dynamics. The investigations of the global stability are essential for understanding the nature and the underlying mechanisms of the game theory dynamics. We show this in an example of repeated Prisoner’s Dilemma game system. This can help the further understanding of the game theory for the real world.

## References

[1] Hofbauer J (2011) Deterministic evolutionary game dynamics. Proceedings of Symposia in Applied Mathematics 69: 61–79. 10.1090/psapm/069/2882634

[2] Sandholm W (2009) Evolutionary Game Theory in Encyclopedia of Complexity and System Science. New York: Springer.

[3] Cason T, Friedman D (2003) Buyer search and price dispersion: A laboratory study. J Econom Theory 112: 232–260. 10.1016/S0022-0531(03)00135-0

[4] Sinervo B, Lively C (1996) The rock-paper-scissors game and the evolution of alternative male strategies. Nature 380: 240–243. 10.1038/380240a0

[5] Maynard S, Price G (1973) The logic of animal conflict. Nature 246: 15–18. 10.1038/246015a0

[6] Nowak M (2004) Evolutionary Dynamics. Cambridge, MA: Harvard University Press.

[7] Foster D, Young P (1990) Stochastic evolutionary game dynamics. Theor Pop Biol 38: 219–232. 10.1016/0040-5809(90)90011-J

[8] Swain P, Elowitz M, Siggia E (2002) Intrinsic and extrinsic contributions to stochasticity in gene expression. Proc Natl Acad Sci USA 99: 12795–12800. 10.1073/pnas.162041399 12237400 PMC130539

[9] Szabo G, Fath G (2007) Evolutionary games on graphs. PhysRep 446: 97–216.

[10] Hommesa C, Ocheab M (2012) Multiple equilibria and limit cycles in evolutionary games with logit dynamics. Games and Economic Behavior 74: 434–441. 10.1016/j.geb.2011.05.014

[11] Hofbauer J, Schuster K, Wolff R (1980) Dynamical systems under constant organization ii: Homogeneous growth functions of degree p = 2. SIAM J Appl Math 38: 282–304. 10.1137/0138025

[12] Zeeman E (1980) Population dynamics from game theory In: Global Theory of Dynamical Systems. New York: Springer.

[13] Pais D, Caicedo-Nuez CH, Leonard NE (2012) Hopf bifurcations and limit cycles in evolutionary network dynamics. Siam Journal on Applied Dynamical Systems 11: 1754–1784. 10.1137/120878537

[14] Weibull J (1995) Evolutionary Game Theory. Cambridge: The MIT press.

[15] Bladon A, Galla T, Mckane A (2010) Evolutionary dynamics, intrinsic noise and cycles of co-operation. Phys Rev E 81: 066122 10.1103/PhysRevE.81.066122 20866493

[16] Allen B, Rosenbloom D (2012) Mutation rate evolution in replicator dynamics. Bull Math Biol 74: 2650–2675. 10.1007/s11538-012-9771-8 22941151

[17] Imhof LA, Fudenberg D, Nowak MA, May RM (2005) Evolutionary cycles of cooperation and defection. Proc Natl Acad Sci USA 102: 10797–10800. 10.1073/pnas.0502589102 16043717 PMC1182423

[18] Toupo DP, Rand DG, Strogatz S (2014) Limit cycles sparked by mutation in the repeated prisoner’s dilemma. International Journal of Bifurcation and Chaos 24: 1430035 10.1142/S0218127414300353

[19] Pais D, Leonard N (2011) Limit cycles in replicator-mutator network dynamics. Decision, Control, European Control Conference 413: 3922–3927. 10.1109/CDC.2011.6160995

[20] Wang J, Xu L, Wang EK (2008) Potential landscape and flux framework of nonequilibrium networks: Robustness, dissipation, and coherence of biochemical oscillations. Proc Natl Acad Sci USA 105: 12271–12276. 10.1073/pnas.0800579105 18719111 PMC2527901

[21] Wang J, Zhang K, Wang EK (2010) Kinetic paths, time scale, and underlying landscapes: A path integral framework to study global natures of nonequilibrium systems and networks. J Chem Phys 133: 125103 10.1063/1.3478547 20886967

[22] Wang J, Li C, Wang E (2010) Potential and flux landscapes quantify the stability and robustness of budding yeast cell cycle network. Proc Natl Acad Sci USA 107: 8195–8200. 10.1073/pnas.0910331107 20393126 PMC2889591

[23] Xu L, Zhang F, Wang EK, Wang J (2013) The potential and flux landscape, lyapunov function and non-equilibrium thermodynamics for dynamic systems and networks with an application to signal-induced ca2+ oscillation. Nonlinearity 26: 69–84. 10.1088/0951-7715/26/2/R69

[24] Zhang F, Xu L, Zhang K, Wang E, Wang J (2012) The potential and flux landscape theory of evolution. J Chem Phys 137: 065102 10.1063/1.4734305 22897313

[25] Xu L, Zhang F, Zhang K, Wang EK, Wang J (2014) The potential and flux landscape theory of ecology. PLoS ONE 9: e86746 10.1371/journal.pone.0086746 24497975 PMC3907570

[26] Xu L, Zhang K, Wang J (2014) Exploring the mechanisms of differentiation, dedifferentiation, reprogramming and transdifferentiation. PLoS ONE 9: e105216 10.1371/journal.pone.0105216 25133589 PMC4136825

[27] Wang J (2015) Landscape and flux theory of non-equilibrium dynamical systems with application to biology. Advances in Physics 64: 1–137. 10.1080/00018732.2015.1037068

[28] Van Kampen NG (2007) Stochastic processes in physics and chemistry. Amsterdam: Elsevier.

[29] Gillespie D (1977) Exact stochastic simulation of coupled chemical reactions. J Phys Chem 81: 2340–2361. 10.1021/j100540a008

[30] Graham R (1989) Macroscopic potentials, bifurcations and noise in dissipative systems In: Moss F, McClintock P, editors, Noise in Nonlinear Dynamical Systems Vol. 1. Cambridge University Press.

[31] Sasai M, Wolynes P (2003) Stochastic gene expression as a many-body problem. Proc Natl Acad Sci USA 100: 2374–2379. 10.1073/pnas.2627987100 12606710 PMC151348

[32] Haken H (1987) Advanced synergetics: instability hierarchies of self-organizing systems and devices. Berlin: Springer.

[33] Hu G (1986) Lyapunov function and stationary probability distributions. Z Phys B: Condens Matter 65: 103–106. 10.1007/BF01308404

[34] Mitchell IM (2008) The flexible, extensible and efficient toolbox of level set methods. J Sci Comput 35: 300–329. 10.1007/s10915-007-9174-4

[35] Wang J, Huang B, Xia XF, Sun ZR (2006) Funneled landscape leads to robustness of cell networks: Yeast cell cycle. PLOS Comp Biol 2: e147 10.1371/journal.pcbi.0020147 PMC1636676 17112311

[36] Ao P (2005) Laws in darwinian evolutionary theory. Physics of Life Reviews 2: 117–156. 10.1016/j.plrev.2005.03.002

[37] Qian H (2009) Entropy demystified: The “thermo”-dynamics of stochastically fluctuating systems. Method Enzymol 467: 111–134. 10.1016/S0076-6879(09)67005-1 19897091

[38] Schnakenberg J (1976) Network theory of microscopic and macroscopic behavior of master equation systems. Rev Mod Phys 48: 571–585. 10.1103/RevModPhys.48.571

[39] Ge H, Qian H (2010) The physical origins of entropy production, free energy dissipation and their mathematical representations. Phys Rev E 81: 051133 10.1103/PhysRevE.81.051133 20866211

[40] Qian H (2006) Open-system nonequilibrium steady-state: Statistical thermodynamics, fluctuations and chemical oscillations. J Phys Chem B 110: 15063–15074. 10.1021/jp061858z 16884217

[41] Rapoport A, Chammah AM (1965) Prisoner’s Dilemma: A Study in Con ict and Cooperation. Ann Arbor: University of Michigan Press.

[42] Danielle FP, Steven HS (2015) Nonlinear dynamics of the rock-paper-scissors game with mutations. Phys Rev E 91: 052907 10.1103/PhysRevE.91.052907 26066229

[43] Taylor P, Jonker L (1978) Evolutionarily stable strategies and game dynamics. Math Biosci 40: 145–156. 10.1016/0025-5564(78)90077-9

[44] Binmore KG, Samuelson L (1991) Evolutionary stability in repeated games played by finite automata. Journal of Economic Theory 57: 278–305. 10.1016/0022-0531(92)90037-I

[45] Axelrod R (1984) The Evolution of Cooperation. New York: Basic Books.

[46] Zhang XP, Liu F, Wang W (2011) Two-phase dynamics of p53 in the DNA damage response. Proc Natl Acad Sci USA 108: 8990–8995. 10.1073/pnas.1100600108 21576488 PMC3107314

